# Activity Detection and Channel Estimation Based on Correlated Hybrid Message Passing for Grant-Free Massive Random Access

**DOI:** 10.3390/e27111111

**Published:** 2025-10-28

**Authors:** Xiaofeng Liu, Xinrui Gong, Xiao Fu

**Affiliations:** 1 School of Artificial Intelligence, Yancheng Teachers University, Yancheng 224000, China; liuxf@yctu.edu.cn; 2 Purple Mountain Laboratories, Nanjing 211100, China; fu_xiao@seu.edu.cn; 3 National Mobile Communications Research Laboratory, Southeast University, Nanjing 210096, China

**Keywords:** activity detection, channel estimation, massive random access, message passing, Bethe free energy

## Abstract

Massive machine-type communications (mMTC) in future 6G networks will involve a vast number of devices with sporadic traffic. Grant-free access has emerged as an effective strategy to reduce the access latency and processing overhead by allowing devices to transmit without prior permission, making accurate active user detection and channel estimation (AUDCE) crucial. In this paper, we investigate the joint AUDCE problem in wideband massive access systems. We develop an innovative channel prior model that captures the dual correlation structure of the channel using three state variables: active indication, channel supports, and channel values. By integrating Markov chains with coupled Gaussian distributions, the model effectively describes both the structural and numerical dependencies within the channel. We propose the correlated hybrid message passing (CHMP) algorithm based on Bethe free energy (BFE) minimization, which adaptively updates model parameters without requiring prior knowledge of user sparsity or channel priors. Simulation results show that the CHMP algorithm accurately detects active users and achieves precise channel estimation.

## 1. Introduction

With the continuous evolution of mobile communication technology and the rapid development of Internet of Things (IoT) applications, future 6G technologies are required to support the massive connectivity of IoT devices. Massive access is integral to emerging fields such as smart homes, smart cities, and industrial automation, with the primary goal of providing ubiquitous connectivity for low-cost, energy-efficient devices [1,2]. However, due to the large scale, sporadic activity, and short data packet transmission characteristic of IoT devices, traditional grant-based random access methods, which rely on negotiation and resource allocation, can lead to heavy signaling overhead and high transmission latency [3]. To address these fundamental challenges, researchers have proposed grant-free access schemes. By allowing devices to transmit data without prior authorization, this paradigm significantly improves the communication efficiency and is considered a cornerstone technology for future massive access scenarios [4].

The successful implementation of grant-free access relies on a critical task at the base station (BS): the ability to efficiently perform joint active user detection and channel estimation (AUDCE) from a single snapshot of superimposed signals [5]. Leveraging the sporadic nature of user activity, this problem can be elegantly formulated as a sparse signal recovery task. Early and foundational approaches were largely based on traditional compressed sensing (CS) algorithms, such as orthogonal matching pursuit (OMP), which provided initial solutions but often exhibited limited performance, especially in low signal-to-noise-ratio (SNR) regimes or when the channel statistics are complex [6,7]. To achieve higher accuracy and robustness, the research paradigm has increasingly shifted towards model-based Bayesian inference, which offers a principled way to incorporate rich prior knowledge of the system’s structure and statistics. Within this paradigm, advanced message passing (MP) algorithms, including those derived from sparse Bayesian learning (SBL) and generalized approximate message passing (GAMP), have emerged as powerful and computationally efficient frameworks for AUDCE, demonstrating significant performance gains over their CS counterparts [8,9,10].

To further push the performance boundaries, a pivotal research direction has been the exploitation of inherent correlation structures within the massive access channel. This body of work can be broadly summarized by the dimension of correlation explored. The core strategy in the time domain is to leverage the temporal dependency of user activity, where a user tends to remain active or inactive over consecutive time slots. Representative works, such as [11,12], have captured this slow-varying behavior using methods like message passing with temporally correlated priors or Bernoulli–Gaussian–Markov chain models. Beyond the time domain, the primary approach in the frequency domain is to mitigate the effects of frequency-selective fading by transforming the channel into a domain where it exhibits a more compact, sparse representation. For instance, the work in [13] effectively exploits channel sparsity in the discrete cosine transform (DCT) domain using a hybrid message passing algorithm. Furthermore, the spatial domain offers yet another layer of rich structural information, where researchers have capitalized on large antenna arrays from two main perspectives. One line of inquiry leverages the channel’s spatial correlation, where works like [14] have incorporated the channel’s spatial covariance matrix into the compressed sensing framework as prior information to enhance the estimation accuracy. Another powerful approach, adopted in [15,16], is to exploit the inherent channel sparsity that emerges when the channel is characterized in the angle domain, which is a direct consequence of the clustered nature of physical scatterers. This research direction has also been successfully extended to next-generation architectures like cell-free massive MIMO systems [17].

Parallel to the advancements in correlation modeling, the research community has explored the AUDCE problem from a diverse set of methodological perspectives, reflecting the vibrancy of this field. A significant recent trend is the rise of data-driven methods; for example, a deep learning-based approach was introduced in [18], which combines a preamble detection neural network and a data detection neural network. Another type of advanced signal processing, namely tensor-based methods, has been utilized to handle more complex, high-dimensional scenarios, such as those involving high user mobility [19]. Furthermore, some works have expanded the problem’s scope to include the subsequent data decoding stage, tackling the entire processing chain from detection to decoding within a unified framework [20]. Concurrently, innovation continues in transmission schemes and problem formulations. Novel techniques like differential modulation have been proposed to simplify detection [21], while others have tackled the more challenging blind access problem in demanding mmWave settings [22] or considered broader system-level requirements such as multi-service provisioning [23].

While the aforementioned works have made significant progress by exploiting individual channel correlations, their focus on a single facet of the channel’s complex statistical topology may be insufficient to provide the full descriptive power needed for the challenging conditions of wideband massive MIMO-OFDM systems. Therefore, a notable avenue for performance enhancement lies in more comprehensive modeling of the channel’s statistical properties. The primary limitation of existing models is their partial exploitation of these properties. They typically focus on the correlation within only one of the channel’s components, such as its structure or its values, while treating the other with a simpler, often independent prior. By not simultaneously modeling both the intricate structural patterns and the nuanced value dependencies, these approaches fail to leverage the full statistical richness of the physical channel. The core challenge, therefore, is to develop a unified probabilistic framework that can cohesively model this dual correlation, which involves capturing both the structured correlation within the channel support and the value correlation within the channel gains, all under a single comprehensive prior. In this paper, we address this critical gap by proposing a novel framework for joint AUDCE under the massive MIMO-OFDM system. The main contributions of our work can be summarized as follows:

- To characterize the sparse properties of the channel in the user–angle–delay domain, we construct a novel hierarchical probabilistic structure that includes three state variables: active indication, channel supports, and channel values. The innovation of this model lies in its layered structure, which provides a principled way to factorize the joint prior distribution and cohesively model sparsity at different levels. This model accurately captures the user-domain sparsity through the active indication variable and, critically, utilizes the channel support and channel values variables to enable the modeling of what we term the dual correlation of user channels. This dual correlation refers to the modeling of two distinct statistical phenomena under a unified prior: the structural correlation, which captures the clustered nature of non-zero channel paths using a Markov chain, and the value correlation, which describes the statistical dependency between the gains of adjacent patterns using a coupled Gaussian distribution.
- Based on the proposed system and probability model, the joint active user detection and channel estimation problem is formulated as a Bethe free energy (BFE) minimization problem under hybrid constraints. Through the optimization and reconstruction of the constraint conditions, we propose the correlated hybrid message passing (CHMP) algorithm. The hybrid nature of the algorithm lies in its tailored message passing schedule, which applies exact inference for discrete state variables (active indication, channel supports) and efficient, moment-based approximate inference for continuous variables (channel values). This approach, derived rigorously under our hybrid constraint framework, achieves a favorable balance between estimation accuracy and computational complexity. Furthermore, this algorithm can adaptively update model parameters without prior knowledge of user sparsity or channel prior information.
- Numerical simulations demonstrate that the proposed joint correlation modeling significantly enhances performance. The reason is that, by exploiting these dual correlations, our algorithm gains powerful prior information that regularizes the ill-posed estimation problem. The structural correlation helps to more accurately locate clusters of active channel taps, while the value correlation helps to refine their coefficient estimates, leading to more robust and precise joint detection and estimation.

To provide a clear overview and roadmap for the reader, we summarize the structure of our proposed solution here. We begin by establishing the system model for a grant-free massive MIMO-OFDM network. Then, to capture the channel’s inherent structure, we develop a novel hierarchical probabilistic prior model that characterizes the dual correlations of channel support via Markov chains and channel values via coupled Gaussian distributions. Based on this model, we formulate the joint estimation problem as a BFE minimization task. Finally, we derive the CHMP algorithm as an efficient iterative solution to this problem, which jointly yields the active user set and their corresponding channels.

The remainder of this paper is organized as follows. In Section 2, the system model is presented for grant-free massive random access in massive MIMO-OFDM systems. In Section 3, the probability model is developed by utilizing three types of state variables—active indicators, channel supports, and channel values—to model the equivalent angle–delay domain channel. Section 4 formulates the joint active user detection and channel estimation problem as a constrained BFE minimization problem. Section 5 provides the detailed theoretical foundation of the CHMP algorithm by solving the constrained BFE minimization problem. Section 6 describes the proposed algorithm, including the procedure details and computational complexity analysis, while Section 7 presents the simulation experiment results. Section 8 concludes this paper.

Notation: The boldface lowercase (uppercase) letters denote vectors (matrices). ${\left(\cdot \right)}^{*}$, ${\left(\cdot \right)}^{T}$, and ${\left(\cdot \right)}^{H}$ denote the matrix conjugate, transpose, and conjugate transpose, respectively. $\left\lfloor \cdot \right\rfloor$, $\ln\left(\cdot \right)$, and Re· denote floor, natural logarithm, and real part operations, respectively. $\delta \left(\cdot \right)$ and $\delta \left[\cdot \right]$ denote continuous and discrete delta functions, respectively. $E\left[\cdot \right]$, $H\left[\cdot \right]$, and $D\left[\cdot \right]$ denote the statistical expectation, entropy, and relative entropy, respectively. ⊗ denotes the Kronecker product, ${∥\cdot ∥}_{2}$ denotes the $\ell_{2}$ norm, ∝ denotes the scalar proportionality between two real-valued functions, and CN(x;μ,υ) denotes the complex Gaussian distribution with mean μ and variance υ. For the reader’s convenience, we summarize the key notations used throughout this paper in Table 1.

## 2. System Model

**Figure 1:**
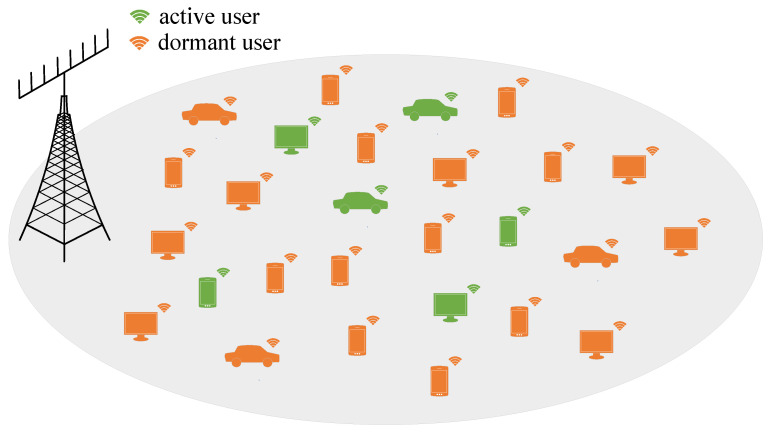
The uplink grant-free random access scenario in a single-cell massive MIMO-OFDM system.

Consider the uplink grant-free random access in a single-cell massive MIMO-OFDM system, as shown in Figure 1. The BS is equipped with a uniform linear array consisting of *M* antennas and serves *K* single-antenna users within the cell. Due to the sporadic transmission characteristic of IoT devices, only a small number of users in the potential user set $K=\left\{1,2,\cdots ,K\right\}$ remain active during uplink access, while the remaining users remain dormant, i.e., $\left|K_{a}\right|=K_{a}\ll K$, where $K_{a}$ denotes the set of active users and $K_{a}\subseteq K$ [24]. This section will first present the channel model for the grant-free massive access system and then provide the received signal model based on the sparse channel representation in the transform domain.

### 2.1. Channel Model

Assume that there are $I_{k}$ propagation paths from user *k* to the BS in the wireless scattering environment. The system employs OFDM modulation with $N_{c}$ subcarriers, where the spacing between adjacent subcarriers is $\Delta_{f}$, and signal transmission occurs over *N* effective subcarriers. The space–frequency domain channel vector for user *k* on the *n*-th effective subcarrier can be expressed as

(1) $$\begin{array}{c} h_{n,k}^{\mathrm{SF}}=\sum_{i=1}^{I_{k}}\beta_{i,k}a\left(\phi_{i,k}\right)e^{-j2\pi f_{n}\tau_{i,k}}, \end{array}$$

where $\beta_{i,k}$ and $\tau_{i,k}$ represent the small-scale complex channel gain and propagation delay of the *i*-th path for user *k*, respectively, and $\phi_{i,k}=\sin\theta_{i,k}$, where $\theta_{i,k}$ is the angle of arrival of the *i*-th path for user *k*. $f_{n}=\left(n-1-\frac{N}{2}\right)\Delta_{f}$ denotes the relative frequency of the *n*-th effective subcarrier with respect to the carrier frequency $f_{c}$, where n=1,⋯,N. The array response vector $a\left(\phi_{i,k}\right)\in C^{M\times 1}$ is given by

(2) $$a\left(\phi_{i,k}\right)={\left[1,e^{-j2\pi \frac{d_{s}}{\lambda }\phi_{i,k}},\cdots ,e^{-j2\pi \left(M-1\right)\frac{d_{s}}{\lambda }\phi_{i,k}}\right]}^{T},$$

where λ represents the wavelength of the carrier, and $d_{s}=\frac{\lambda }{2}$ denotes the half-wavelength antenna spacing. Thus, the space–frequency domain channel matrix for user *k* can be constructed from its space–frequency domain channel vectors of the effective subcarriers as follows:

(3) $$\begin{array}{cc} H_{k}^{\mathrm{SF}} & =\left[h_{1,k}^{\mathrm{SF}},h_{2,k}^{\mathrm{SF}},\cdots ,h_{N,k}^{\mathrm{SF}}\right]\in C^{M\times N}\\ \; & =\sum_{i=1}^{I_{k}}\beta_{i,k}a\left(\phi_{i,k}\right)b{\left(\tau_{i,k}\right)}^{T}, \end{array}$$

where the delay response vector $b\left(\tau_{i,k}\right)\in C^{N\times 1}$ is given by   

(4) $$b\left(\tau_{i,k}\right)={\left[e^{-j2\pi f_{1}\tau_{i,k}},e^{-j2\pi f_{2}\tau_{i,k}},\cdots ,e^{-j2\pi f_{N}\tau_{i,k}}\right]}^{T}.$$

Then, by implementing quantization sampling in the angle and delay domains, we can establish the transformation relationship between the space–frequency domain channel and the angle–delay domain channel. Since $\phi_{i,k}=\sin\theta_{i,k}$, its value ranges within $\left[-1,\;1\right]$. Assuming that the cyclic prefix (CP) length of the OFDM system is $N_{\mathrm{cp}}$ system sampling intervals, which exceeds the maximum path delay, the value range of $\tau_{i,k}$ is $\left[0,\;N_{\mathrm{cp}}T_{s}\right]$, where $T_{s}=\frac{1}{N_{c}\Delta_{f}}$ is the system sampling interval. Consider uniformly sampling the value ranges of $\phi_{i,k}$ and $\tau_{i,k}$ using *D* and *L* grid points, respectively. To ensure sufficient quantization accuracy, the conditions D≥M and $L\geq N_{\mathrm{cp}}$ should be satisfied. Therefore, the sampled angle $\phi_{d}$ and sampled delay $\tau_{l}$ can be expressed as

(5) $$\begin{array}{cc} \; & \phi_{d}=\frac{2}{D}\left(d-1\right)-1,\;d=1,2,\cdots ,D, \end{array}$$

(6) $$\begin{array}{cc} \; & \tau_{l}=\frac{N_{\mathrm{cp}}}{LN_{c}\Delta_{f}}\left(l-1\right),\;\;l=1,2,\cdots ,L. \end{array}$$

Next, we can construct the angle domain sampling matrix A and the delay domain sampling matrix B, which are composed of their respective sampling response vectors and are specifically given by

(7) $$\begin{array}{cc} \; & A=\left[a\left(\phi_{1}\right),a\left(\phi_{2}\right),\cdots ,a\left(\phi_{D}\right)\right]\in C^{M\times D}, \end{array}$$

(8) $$\begin{array}{cc} \; & B=\left[b\left(\tau_{1}\right),b\left(\tau_{2}\right),\cdots ,b\left(\tau_{L}\right)\right]\in C^{N\times L}. \end{array}$$

With the angle and delay domain sampling matrices A and B now defined, we can express the physical space–frequency channel $H_{k}^{\mathrm{SF}}$ in terms of its angle–delay domain counterpart, which we denote as $H_{k}^{\mathrm{AD}}\in C^{D\times L}$. Therefore, the space–frequency domain channel matrix $H_{k}^{\mathrm{SF}}$ in (3) can be re-expressed as

(9) $$\begin{array}{c} H_{k}^{\mathrm{SF}}={\mathrm{AH}}_{k}^{\mathrm{AD}}B^{T}. \end{array}$$

### 2.2. Received Signal Model

Define the active indicator factor $\alpha_{k}\in \left\{0,1\right\}$ to denote the active status of the user *k*. When $k\in K_{a}$, we have $\alpha_{k}=1$, indicating that the user is active; conversely, when $k\in K\setminus K_{a}$, we have $\alpha_{k}=0$, indicating that the user is dormant. Thus, the received signal at the BS for the *t*-th OFDM symbol can be given by

(10) $$\begin{array}{cc} {\tilde{Y}}_{t} & =\sum_{k=1}^{K}\alpha_{k}\sqrt{p_{k}\chi_{k}}H_{k}^{\mathrm{SF}}{\tilde{X}}_{k,t}+{\tilde{N}}_{t}\\ \; & =\sum_{k=1}^{K}\sqrt{{\tilde{\chi }}_{k}}H_{k}^{\mathrm{sf}}{\tilde{X}}_{k,t}+{\tilde{N}}_{t}, \end{array}$$

where ${\tilde{Y}}_{t}\in C^{M\times N}$, $H_{k}^{\mathrm{sf}}=\alpha_{k}H_{k}^{\mathrm{SF}}$ represents the equivalent space–frequency domain channel for user *k*; $p_{k}$ and $\chi_{k}$ denote the signal transmission power and large-scale fading factor of user *k* when active, respectively; and ${\tilde{\chi }}_{k}=p_{k}\chi_{k}$ represents the equivalent large-scale fading factor for user *k*. ${\tilde{X}}_{k,t}=\mathrm{diag}\left({\tilde{x}}_{k,t}\right)\in C^{N\times N}$ denotes the unit energy transmitted signal of the user *k*, satisfying the power constraint $E\left[{\tilde{x}}_{k,t}{\tilde{x}}_{k,t}^{H}\right]=I$, and ${\tilde{N}}_{t}$ represents the additive white Gaussian noise matrix with zero mean and variance σ. Assuming effective power control, each user’s signal transmission power can compensate for channel large-scale fading, resulting in equal equivalent large-scale factors for all users [11,25], i.e., ${\tilde{\chi }}_{k}=\chi$, ∀k. To simplify the discussion, let χ=1. The small-scale channel matrix $H_{k}^{\mathrm{SF}}$ is assumed to have the same power, i.e., $E\left[∥H_{k}^{\mathrm{SF}}{∥}_{F}^{2}\right]=\xi$, ∀k. This ensures that the total received signal power from each active user in the BS is equal, avoiding the impact of the near–far effect on the users [26]. Finally, the equivalent space–frequency domain channel matrix for all users is defined as $H^{\mathrm{sf}}=\left[H_{1}^{\mathrm{sf}},H_{2}^{\mathrm{sf}},\cdots ,H_{K}^{\mathrm{sf}}\right]\in C^{M\times NK}$.

Assume that all users transmit uplink access pilots using the same subcarrier set P when active, with the number of pilot subcarriers being *P*, embedded uniformly in the *N* effective subcarriers. The system assigns a unique Gaussian access pilot sequence $\left\{x_{k,t}|t\in T\right\}$ to each user, where $T=\left\{t_{1},t_{2},\cdots ,t_{G}\right\}$ represents the set of *G* OFDM symbols used for access, and $x_{k,t}\in C^{P\times 1}$, with elements $x_{p,k,t}$ following the standard complex Gaussian distribution $\mathrm{CN}(x_{p,k,t};0,1)$. Then, the received pilot signal at the BS for the *t*-th OFDM symbol, t∈T, can be expressed as

(11) $$\begin{array}{cc} Y_{t} & =\sum_{k=1}^{K}H_{k}^{\mathrm{sf}}{\tilde{X}}_{k,t}\Xi +N_{t}\\ \; & =A\sum_{k=1}^{K}H_{k}^{\mathrm{ad}}B^{T}\Xi X_{k,t}+N_{t}\\ \; & =AH^{\mathrm{ad}}\left(I_{K}⊗\left(B^{T}\Xi \right)\right)X_{t}+N_{t}, \end{array}$$

where $\Xi \in {\left\{0,1\right\}}^{N\times P}$ is the index matrix for the pilot subcarriers, composed of columns from $I_{N}$ corresponding to P. $Y_{t}={\tilde{Y}}_{t}\Xi \in C^{M\times P}$ and $N_{t}={\tilde{N}}_{t}\Xi \in C^{M\times P}$ represent the received pilot signal and noise matrix, respectively. $X_{k,t}=\mathrm{diag}\left(x_{k,t}\right)\in C^{P\times P}$, and ${\tilde{X}}_{k,t}\Xi =\Xi X_{k,t}$. $H_{k}^{\mathrm{ad}}=\alpha_{k}H_{k}^{\mathrm{AD}}$ is the equivalent angle–delay domain channel matrix for user *k*, $H^{\mathrm{ad}}=\left[H_{1}^{\mathrm{ad}},H_{2}^{\mathrm{ad}},\cdots ,H_{K}^{\mathrm{ad}}\right]\in C^{D\times LK}$ is the equivalent angle–delay domain channel matrix for all users, and $X_{t}={\left[X_{1,t}^{T},X_{2,t}^{T},\cdots ,X_{K,t}^{T}\right]}^{T}\in C^{PK\times P}$ is the pilot matrix for the *t*-th OFDM symbol. Therefore, the overall received pilot signal at the BS can be formulated as

(12) $$\begin{array}{cc} Y & =AH^{\mathrm{ad}}\left(I_{K}⊗\left(B^{T}\Xi \right)\right)X+N\\ \; & =AH^{\mathrm{ad}}\tilde{B}+N, \end{array}$$

where $Y=\left[Y_{t_{1}},Y_{t_{2}},\cdots ,Y_{t_{G}}\right]\in C^{M\times PG}$, $X=\left[X_{t_{1}},X_{t_{2}},\cdots ,X_{t_{G}}\right]\in C^{PK\times PG}$, $N=\left[N_{t_{1}},N_{t_{2}},\cdots ,N_{t_{G}}\right]\in C^{M\times PG}$, and $\tilde{B}=\left(I_{K}⊗\left(B^{T}\Xi \right)\right)X\in C^{LK\times PG}$. By vectorizing the received signal Y, (12) can be further transformed into the following matrix–vector multiplication form:

(13) $$\begin{array}{c} y=\left({\tilde{B}}^{T}⊗A\right)h+n=\Phi h+n, \end{array}$$

where $y=\mathrm{vec}\left(Y\right)\in C^{R\times 1}$, $h=\mathrm{vec}\left(H^{\mathrm{ad}}\right)\in C^{J\times 1}$, $n=\mathrm{vec}\left(N\right)\in C^{R\times 1}$, and $\Phi ={\tilde{B}}^{T}⊗A\in C^{R\times J}$ are the sensing matrices, where R=MPG and J=DLK. To simplify subsequent notations, we define $r=\left(g-1\right)MP+\left(p-1\right)M+m$ and $j=\left(k-1\right)DL+\left(l-1\right)D+d$ and also define the subscript ${\left(\cdot \right)}_{i}={\left(\cdot \right)}_{m,p,g}$ and ${\left(\cdot \right)}_{j}={\left(\cdot \right)}_{d,l,k}$. It is important to note that, while we assume unique Gaussian pilots, this choice serves as a standard model for the non-orthogonal pilot scenario common in massive access systems [20,23,27] and is widely adopted for its analytical tractability. The specific properties of the pilot sequences are entirely captured within the sensing matrix Φ. Therefore, the algorithm subsequently proposed in this paper is a general framework that is not restricted to a specific pilot type.

The goal of this paper is to jointly detect the activity of all users $\left\{\alpha_{k}|k\in K\right\}$ and estimate the space–frequency domain channels of active users $\left\{H_{k}^{\mathrm{SF}}|k\in K_{a}\right\}$ using the pilot reception signal y from the BS and the received signal model (13). Since the user’s activity status is embedded in the equivalent channel, this target is equivalent to estimating the equivalent space–frequency domain channels of all users $\left\{H_{k}^{\mathrm{sf}}|k\in K\right\}$ [9]. Furthermore, based on the domain transformation relationship of the channel (9), the space–frequency domain channels $\left\{H_{k}^{\mathrm{sf}}|k\in K\right\}$ can be reconstructed by obtaining the equivalent angle–delay domain channels $\left\{H_{k}^{\mathrm{ad}}|k\in K\right\}$.

## 3. Probability Model

This section establishes a probability model to characterize the active sparsity of the equivalent channel in the user dimension and the sparse correlation in the angle–delay domain. Based on the previous system model, we first provide a probability representation of the received signal model. Then, the prior probabilities of the equivalent angle–delay domain channel are modeled using three types of state variables: active indicators, channel support, and channel values. The sparse correlation between channel support and channel values is detailed using a combination of Markov chains and coupled Gaussian distributions. Finally, the corresponding posterior probability representation is constructed for the joint problem of active user detection and channel estimation.

### 3.1. Received Signal Probability Formulation

Based on the received signal model (13), we begin by expressing the problem in a probabilistic form. The likelihood of the received signal given the channel can be written as a marginalization over an auxiliary noiseless signal variable z:

(14) $$\begin{array}{c} p\left(y|h\right)=\int p\left(y|z\right)p\left(z|h\right)dz, \end{array}$$

where the auxiliary vector $z=\Phi h\in C^{R\times 1}$ represents the BS’s received pilot signal in the absence of noise. Specifically, the conditional probability density $p\left(y|z\right)$ can be expressed as

(15) $$\begin{array}{c} p\left(y|z\right)=\prod_{r=1}^{R}\underset{\overset{\Delta }{\;=}f_{Y,r}\left(z_{r}\right)}{\underset{︸}{p\left(y_{r}|z_{r}\right)}}=\prod_{r=1}^{R}\mathrm{CN}\left(y_{r};z_{r},\sigma \right), \end{array}$$

where $y_{r}$ and $z_{r}$ represent the *r*-th elements of y and z, respectively, and $f_{Y,r}\left(z_{r}\right)$ denotes the corresponding factor function form, provided here for ease of subsequent discussion. The conditional probability density $p\left(z|h\right)$ is specifically given by

(16) $$\begin{array}{c} p\left(z|h\right)=\prod_{r=1}^{R}\underset{\overset{\Delta }{\;=}f_{Z,r}\left(z_{r},h\right)}{\underset{︸}{p\left(z_{r}|h\right)}}=\prod_{r=1}^{R}\delta \left(z_{r}-\sum_{j=1}^{J}\varphi_{r,j}h_{j}\right), \end{array}$$

where $\varphi_{r,j}$ denotes the (r,j)-th element of Φ, $h_{j}$ denotes the *j*-th element of h, and $\delta \left(\cdot \right)$ denotes the continuous Dirac delta function. This representation is a crucial modeling choice with a threefold purpose. Fundamentally, it embeds the deterministic linear constraint z=Φh into the probabilistic factor graph. Structurally, this factorization allows the global problem to be decomposed, which facilitates the modular derivation of our algorithm. Most importantly, it is a prerequisite for the subsequent constraint reconstruction within the BFE framework, where the belief associated with this factor is relaxed.

### 3.2. Prior Model of the Equivalent Channel

To reveal the sparsity characteristic of the equivalent angle–delay domain channel $\left\{H_{k}^{\mathrm{ad}}=\alpha_{k}H_{k}^{\mathrm{AD}},k\in K\right\}$ in terms of users, angles, and delays, we further construct the corresponding prior probability models. To model the channel’s inherent sparse structure in the angle–delay domain, we decompose the physical channel matrix into its structural and numerical components using two types of latent state matrices:

(17) $$\begin{array}{c} H_{k}^{\mathrm{AD}}=S_{k}⊙G_{k}, \end{array}$$

where the channel support $S_{k}\in {\left\{0,1\right\}}^{D\times L}$ is a binary matrix designed to describe the sparse structure of the channel; the channel value $G_{k}\in C^{D\times L}$ is a complex matrix representing the numerical response of the channel. Specifically, the element $s_{d,l,k}$ of $S_{k}$ indicates whether a particular channel element in $H_{k}^{\mathrm{AD}}$ is zero, while the element $g_{d,l,k}$ of $G_{k}$ is a complex-valued random variable representing the channel gain of the corresponding non-zero path. Here, the indices (d,l,k) correspond to the *d*-th angle grid point, the *l*-th delay grid point, and the *k*-th user, respectively. Thus, given $\alpha_{k}$, $S_{k}$, and $G_{k}$, the conditional probability density of the equivalent channel $H_{k}^{\mathrm{ad}}$ is expressed as

(18) $$\begin{array}{cc} p\left(H_{k}^{\mathrm{ad}}|\alpha_{k},S_{k},G_{k}\right) & =\prod_{d=1}^{D}\prod_{l=1}^{L}\underset{\overset{\Delta }{\;=}f_{H,d,l,k}\left(h_{d,l,k},\alpha_{k},\;s_{d,l,k},\;g_{d,l,k}\right)}{\underset{︸}{p\left(h_{d,l,k}|\alpha_{k},s_{d,l,k},g_{d,l,k}\right)}}\\ \; & =\prod_{d=1}^{D}\prod_{l=1}^{L}\delta \left(h_{d,l,k}-\alpha_{k}\;s_{d,l,k}\;g_{d,l,k}\right), \end{array}$$

in which $G_{k}$ and $S_{k}$ are mutually independent. Next, we will establish probability models for these latent variables separately to fully leverage their sparse characteristics.

The user-domain sparsity is captured by the activity indicator $\alpha_{k}$, which is modeled as a Bernoulli random variable with the following prior probability:

(19) $$\begin{array}{c} p\left(\alpha_{k};\rho_{k}\right)\overset{\Delta }{\;=}f_{A,k}\left(\alpha_{k}\right)={\rho_{k}}^{\alpha_{k}}{\left(1-\rho_{k}\right)}^{1-\alpha_{k}}, \end{array}$$

where $\rho_{k}$ denotes the activity probability of user *k*. Considering the practical finite scattering environment, the angle–delay domain channel exhibits a clustered sparse structure, with the non-zero elements of $S_{k}$ primarily concentrated in specific angles corresponding to a limited number of scattering paths. Therefore, a Markov chain can be used to capture the clustered characteristics in the angle domain for each path delay. The prior distribution of the channel support $S_{k}$ can be given by

(20) $$\begin{array}{c} p\left(S_{k};\rho_{k}^{0,1},\rho_{k}^{1,0}\right)=\prod_{l=1}^{L}\left(\underset{\overset{\Delta }{\;=}f_{S,1,l,k}\left(s_{1,l,k}\right)}{\underset{︸}{p\left(s_{1,l,k};\rho_{l,k}^{0,1},\rho_{l,k}^{1,0}\right)}}\prod_{d=2}^{D}\underset{\overset{\Delta }{\;=}f_{S,d,l,k}\left(s_{d,l,k},s_{d-1,l,k}\right)}{\underset{︸}{p\left(s_{d,l,k}|s_{d-1,l,k};\rho_{l,k}^{0,1},\rho_{l,k}^{1,0}\right)}}\right), \end{array}$$

where $\rho_{l,k}^{0,1}$ denotes the transition probability from 1 to 0, and $\rho_{l,k}^{1,0}$ denotes the transition probability from 0 to 1. $\rho_{k}^{0,1}={\left[\rho_{1,k}^{0,1},\rho_{2,k}^{0,1},\cdots ,\rho_{L,k}^{0,1}\right]}^{T}$ and $\rho_{k}^{1,0}={\left[\rho_{1,k}^{1,0},\rho_{2,k}^{1,0},\cdots ,\rho_{L,k}^{1,0}\right]}^{T}$. For d=2,⋯,D, the conditional probability distribution $p\left(s_{d,l,k}|s_{d-1,l,k};\rho_{l,k}^{0,1},\rho_{l,k}^{1,0}\right)$ is specifically written as

(21) $$\begin{array}{cc} p\left(s_{d,l,k}|s_{d-1,l,k};\rho_{l,k}^{0,1},\rho_{l,k}^{1,0}\right) & ={\left(1-\rho_{l,k}^{0,1}\right)}^{s_{d,l,k}s_{d-1,l,k}}{\left(\rho_{l,k}^{0,1}\right)}^{\left(1-s_{d,l,k}\right)s_{d-1,l,k}}\\ \; & \times {\left(\rho_{l,k}^{1,0}\right)}^{s_{d,l,k}\left(1-s_{d-1,l,k}\right)}{\left(1-\rho_{l,k}^{1,0}\right)}^{\left(1-s_{d,l,k}\right)\left(1-s_{d-1,l,k}\right)}. \end{array}$$

When d=1, the initial probability distribution $p\left(s_{1,l,k};\rho_{l,k}^{0,1},\rho_{l,k}^{1,0}\right)$ is modeled as the steady-state distribution of the Markov chain:

(22) $$\begin{array}{c} p\left(s_{1,l,k};\rho_{l,k}^{0,1},\rho_{l,k}^{1,0}\right)=\frac{1}{\rho_{l,k}^{0,1}+\rho_{l,k}^{1,0}}{\left(\rho_{l,k}^{1,0}\right)}^{s_{1,l,k}}{\left(\rho_{l,k}^{0,1}\right)}^{1-s_{1,l,k}}. \end{array}$$

Finally, to describe the correlation characteristics of adjacent elements in the channel values $G_{k}$, a coupled Gaussian distribution is used for modeling, which is given by

(23) $$\begin{array}{cc} p\left(G_{k}|\Omega_{k}\right) & =\prod_{d=1}^{D}\prod_{l=1}^{L}\underset{\overset{\Delta }{\;=}f_{G,d,l,k}\left(g_{d,l,k},\omega_{d,l,k}\right)}{\underset{︸}{p\left(g_{d,l,k}|\omega_{d,l,k}\right)}}\\ \; & =\prod_{d=1}^{D}\prod_{l=1}^{L}\mathrm{CN}\left(g_{d,l,k};0,{\left(\sum_{\left(d^{\prime },l^{\prime },k\right)\in N_{d,l,k}}\omega_{d^{\prime },l^{\prime },k}\right)}^{-1}\right), \end{array}$$

where $\omega_{d,l,k}$ represents the latent precision of the coupled Gaussian distribution, and $\Omega_{k}\in R^{D\times L}$ is a latent precision matrix aligned with the element positions of $G_{k}$. The set $N_{d,l,k}$ defines the nearest neighbors and the index of $\omega_{d,l,k}$ in the angle–delay domain, and $\omega_{d,l,k}$ represents the vector consisting of $\omega_{d,l,k}$ and its nearest neighbors. Specifically, when the coordinate of $\omega_{d,l,k}$ is inside the angle–delay domain, i.e., 2≤d≤D−1 and 2≤l≤L−1, we have $N_{d,l,k}=\left\{\left(d,l,k\right),\left(d-1,l,k\right),\left(d+1,l,k\right),\left(d,l-1,k\right),\left(d,l+1,k\right)\right\}$ and $\omega_{d,l,k}={\left[\omega_{d,l,k},\omega_{d-1,l,k},\omega_{d+1,l,k},\omega_{d,l-1,k},\omega_{d,l+1,k}\right]}^{T}$. When the coordinate of $\omega_{d,l,k}$ is on the edge or corner of the angle–delay domain, the definitions of $N_{d,l,k}$ and $\omega_{d,l,k}$ need to be adjusted accordingly. This indicates that the channel value $g_{d,l,k}$ shares the latent precision $\omega_{d,l,k}$ with its neighboring elements. The coupling connection of the latent precision illustrates the statistical correlation between channel value responses. Furthermore, the latent precision matrix $\Omega_{k}$ is further modeled as the following Gamma distribution:

(24) $$\begin{array}{cc} p\left(\Omega_{k}\right) & =\prod_{d=1}^{D}\prod_{l=1}^{L}\underset{\overset{\Delta }{\;=}f_{\Omega ,d,l,k}\left(\omega_{d,l,k}\right)}{\underset{︸}{p\left(\omega_{d,l,k}\right)}}=\prod_{d=1}^{D}\prod_{l=1}^{L}\mathrm{Ga}\left(\omega_{d,l,k};a,b\right)\\ \; & =\prod_{d=1}^{D}\prod_{l=1}^{L}\frac{b^{a}}{\Gamma \left(a\right)}\omega_{d,l,k}^{a-1}e^{-b\omega_{d,l,k}}, \end{array}$$

where Γ(·) denotes the Gamma function, and *a* and *b* are preset hyperparameters corresponding to the shape parameter and rate parameter, respectively. When prior knowledge about random variables is lacking, *a* and *b* are often set to very small positive numbers close to zero, such as $10^{-20}$, to ensure that the prior does not contain any information [28].

We note that a similar probabilistic structure, which also employs a Bernoulli–Gaussian process and a Markov chain, has been considered in [12] for grant-free NOMA systems. However, there are several fundamental distinctions between our proposed dual correlation model and the model presented in that work. First, our model is tailored to the massive MIMO-OFDM system and thus operates in the user–angle–delay domain, whereas the previous work was focused on a single-antenna BS system. Second, and more critically, the application of the Markov chain is fundamentally different. We employ it to capture the spatial structural correlation of the channel supports across the angular dimension for each delay tap. In contrast, the referenced work uses it to model the temporal correlation of the binary user activity state across time slots. Third, our model introduces a second, distinct layer of correlation by capturing the intrinsic dependency between adjacent channel patterns via a coupled Gaussian distribution, a component that is absent in the model from the other work. Fourth, our primary objective is the fundamental problem of AUDCE, while the objective in the referenced work is data detection. In summary, by jointly modeling both spatial structural and numerical value correlations for a distinct problem, our proposed framework provides a more comprehensive and accurate prior for the complex channels found in wideband massive MIMO systems.

### 3.3. Probability Representation of the Target Problem

Based on the probability models provided above, the joint posterior probability density function of the equivalent channel, activity indicator, channel support, and channel value can be decomposed as follows:

(25) $$\begin{array}{cc} \; & p\left(z,h,\alpha ,s,g,\omega |y;G\right)=Z^{-1}p\left(y|z\right)p\left(z|h\right)\\ \; & \times \prod_{k=1}^{K}\left(p\left(H_{k}|\alpha_{k},S_{k},G_{k}\right)p\left(\alpha_{k};\rho_{k}\right)p\left(S_{k};\rho_{k}^{0,1},\rho_{k}^{1,0}\right)p\left(G_{k}|\Omega_{k}\right)p\left(\Omega_{k}\right)\right), \end{array}$$

where *Z* is a normalization constant; $\alpha ={\left[\alpha_{1},\alpha_{2},\cdots ,\alpha_{K}\right]}^{T}$, $s=\mathrm{vec}\left(\left[S_{1},S_{2},\cdots ,S_{K}\right]\right)$, $g=\mathrm{vec}\left(\left[G_{1},G_{2},\cdots ,G_{K}\right]\right)$, $\omega =\mathrm{vec}\left(\left[\Omega_{1},\Omega_{2},\cdots ,\Omega_{K}\right]\right)$, and $G=\left\{\rho_{k},\rho_{k}^{0,1},\rho_{k}^{1,0}|k\in K\right\}$ represent the set of model parameters. The joint active user detection and channel estimation can be approached by obtaining the equivalent channel. Based on (25), the minimum mean square error (MMSE) estimator of the equivalent channel $h_{d,l,k}$ can be given by

(26) $$\begin{array}{c} {\hat{h}}_{d,l,k}=\int h_{d,l,k}\;p\left(h_{d,l,k}|y;G\right)dh_{d,l,k}, \end{array}$$

and the marginal posterior probability density $p\left(h_{d,l,k}|y;G\right)$ can be specifically expressed as

(27) $$\begin{array}{c} p\left(h_{d,l,k}|y;G\right)=\int \sum_{\alpha ,s}p\left(z,h,\alpha ,s,g,\omega |y;G\right)dzdgd\omega dh_{\setminus h_{d,l,k}}. \end{array}$$

**Figure 2:**
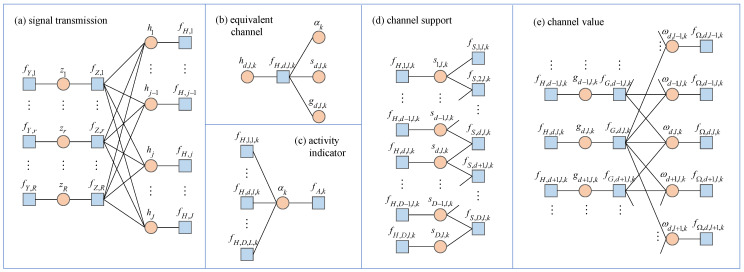
The factor graph of $p\left(z,h,\alpha ,s,g,\omega |y;G\right)$ in (25).

To clearly demonstrate the relationships between the probabilities, we visualize the factorization of the joint posterior probability $p\left(z,h,\alpha ,s,g,\omega |y;G\right)$ in (25), as shown in Figure 2, where blue squares represent factor nodes, while orange circles represent variable nodes. The factor graph in Figure 2 comprises five main modules, corresponding to signal transmission, equivalent channels, activity indications, channel support, and channel values.

Due to the large number of potential users, the factor graph exhibits a complex multi-loop structure, requiring that the exact calculation of the MMSE estimator of the equivalent channel $h_{d,l,k}$ in (26) involves high-dimensional integrals, which are difficult to handle. The next section will transform this complex probability density function integral into a solvable optimization problem based on the constrained BFE minimization theory, combining Bethe approximation and constraint reconstruction methods, thereby laying a solid theoretical foundation for subsequent algorithm design.

## 4. Constrained BFE Minimization Problem

This section will transform the high-dimensional probability density integral calculation into a constrained BFE minimization problem, laying the theoretical foundation for the hybrid message passing algorithm. First, the original BFE expression for the proposed probability model will be established using the Bethe approximation method. Next, a series of constraints on the beliefs of the free energy will be reconstructed to facilitate algorithm design. Finally, the constrained BFE minimization problem will be formulated.

### 4.1. Free Energy and Bethe Approximation

To calculate the marginal posterior probability density $p\left(h_{d,l,k}|y;G\right)$, we first consider the joint posterior probability density $p\left(z,h,\alpha ,s,g,\omega |y;G\right)$. To solve for the marginal posterior probabilities, we turn to the framework of variational inference. According to the definition provided in [29,30,31], the variational free energy is given by

(28) $$\begin{array}{cc} F_{V}\left(b\right) & =\int \sum_{\alpha ,s}b\left(z,h,\alpha ,s,g,\omega \right)\ln\frac{b\left(z,h,\alpha ,s,g,\omega \right)}{p\left(z,h,\alpha ,s,g,\omega ,y;G\right)}dzdhdgd\omega \\ \; & =D\left[b\left(z,h,\alpha ,s,g,\omega \right)∥\;p\left(z,h,\alpha ,s,g,\omega |y;G\right)\right]-\ln Z, \end{array}$$

where lnZ is the normalization constant. Directly minimizing this free energy is intractable. Therefore, we approximate the true belief with the Bethe approximation, which factorizes the belief $b\left(z,h,\alpha ,s,g,\omega \right)$ according to the structure of the factor graph:

(29) $$\begin{array}{cc} b\left(z,h,\alpha ,s,g,\omega \right)=\underset{\mathrm{signal}\;\mathrm{transmission}}{\underset{︸}{\prod_{r=1}^{R}\frac{b_{Y,r}\left(z_{r}\right)b_{Z,r}\left(z_{r},h\right)}{q_{Z,r}\left(z_{r}\right)}}}\;\underset{\mathrm{equivalent}\;\mathrm{channel}}{\underset{︸}{\prod_{j=1}^{J}\frac{b_{H,j}\left(h_{j},\alpha_{k},s_{j},g_{j}\right)}{{\left(q_{H,j}\left(h_{j}\right)\right)}^{R}}}}\;\underset{\mathrm{activity}\;\mathrm{indicator}}{\underset{︸}{\prod_{k=1}^{K}\frac{b_{A,k}\left(\alpha_{k}\right)}{{\left(q_{A,k}\left(\alpha_{k}\right)\right)}^{DL}}}} & \\ \times \underset{\mathrm{channel}\;\mathrm{support}}{\underset{︸}{\prod_{l=1}^{L}\prod_{k=1}^{K}\frac{b_{S,1,l,k}\left(s_{1,l,k}\right)\prod_{d=2}^{D}b_{S,d,l,k}\left(s_{d,l,k},s_{d-1,l,k}\right)}{q_{S,D,l,k}\left(s_{D,l,k}\right)\prod_{d=1}^{D-1}{\left(q_{S,d,l,k}\left(s_{d,l,k}\right)\right)}^{2}}}}\;\underset{\mathrm{channel}\;\mathrm{value}}{\underset{︸}{\prod_{j=1}^{J}\frac{b_{G,j}\left(g_{j},\omega_{j}\right)b_{\Omega ,j}\left(\omega_{j}\right)}{q_{G,j}\left(g_{j}\right){\left(q_{\Omega ,j}\left(\omega_{j}\right)\right)}^{\left|N_{j}\right|}}}} & , \end{array}$$

where $\left|N_{j}\right|$ denotes the cardinality of the set $N_{j}$. The factor beliefs and variable beliefs can also be divided into five categories, corresponding to signal transmission, equivalent channels, activity indicators, channel support, and channel values, as listed in Table 2.

Moreover, the Bethe approximation requires that the factor beliefs and variable beliefs satisfy the following marginal consistency constraints:

(30) $$\begin{array}{cc} \; & q_{Z,r}\left(z_{r}\right)=b_{Y,r}\left(z_{r}\right)=\int b_{Z,r}\left(z_{r},h\right)dh, \end{array}$$

(31) $$\begin{array}{cc} \; & q_{H,j}\left(h_{j}\right)=\int b_{Z,r}\left(z_{r},h\right)dz_{r}dh_{\setminus h_{j}}=\int \sum_{\alpha_{k},s_{j}}b_{H,j}\left(h_{j},\alpha_{k},s_{j},g_{j}\right)dg_{j}, \end{array}$$

(32) $$\begin{array}{cc} \; & q_{A,k}\left(\alpha_{k}\right)=b_{A,k}\left(\alpha_{k}\right)=\int \sum_{s_{j}}b_{H,j}\left(h_{j},\alpha_{k},s_{j},g_{j}\right)dh_{j}dg_{j}, \end{array}$$

(33) $$\begin{array}{cc} \; & q_{G,j}\left(g_{j}\right)=\int b_{G,j}\left(g_{j},\omega_{j}\right)d\omega_{j}\;=\int \sum_{\alpha_{k},s_{j}}b_{H,j}\left(h_{j},\alpha_{k},s_{j},g_{j}\right)dh_{j}, \end{array}$$

(34) $$\begin{array}{cc} \; & q_{\Omega ,j}\left(\omega_{j}\right)=b_{\Omega ,j}\left(\omega_{j}\right)=\int b_{G,j^{\prime }}\left(g_{j^{\prime }},\omega_{j^{\prime }}\right)dg_{j^{\prime }}d\omega_{j^{\prime }\setminus j},\;j^{\prime }\in N_{j}, \end{array}$$

(35) $$\begin{array}{cc} q_{S,d,l,k}\left(s_{d,l,k}\right) & =\int \sum_{\alpha_{k}}b_{H,d,l,k}\left(h_{d,l,k},\alpha_{k},s_{d,l,k},g_{d,l,k}\right)dh_{d,l,k}dg_{d,l,k}\\ & =\sum_{s_{d+1,l,k}}b_{S,d+1,l,k}\left(s_{d+1,l,k},s_{d,l,k}\right),\;d=1,\cdots ,D-1,\\ & =\left\{\begin{array}{c} b_{S,1,l,k}\left(s_{1,l,k}\right),\;\;\;\\ \sum_{s_{d-1,l,k}}b_{S,d,l,k}\left(s_{d,l,k},s_{d-1,l,k}\right),\;d=2,\cdots ,D, \end{array}\right. \end{array}$$

where the constraint (35) uses three-dimensional coordinates $\left(d,l,k\right)$ to clearly demonstrate the relationship between the beliefs. Additionally, the factor beliefs and variable beliefs need to satisfy the following normalization constraints:

(36) $$\begin{array}{cc} \; & E_{b_{Y,r}}\left[1\right]=E_{q_{Z,r}}\left[1\right]=E_{b_{Z,r}}\left[1\right]=E_{q_{H,j}}\left[1\right]=E_{b_{H,j}}\left[1\right]=E_{q_{A,k}}\left[1\right]\\ = & E_{b_{A,k}}\left[1\right]=E_{q_{S,j}}\left[1\right]=\;E_{b_{S,j}}\left[1\right]=E_{q_{G,j}}\left[1\right]=E_{b_{\Omega ,j}}\left[1\right]=1, \end{array}$$

(37) $$\begin{array}{cc} \; & E_{b_{G,j}}\left[1\right]=E_{q_{\Omega ,j}}\left[1\right]=1, \end{array}$$

where (37) is listed separately to facilitate the discussion in the next subsection. Since the marginal consistency constraints and normalization constraints ensure that the non-negativity constraints of the beliefs are always satisfied, their non-negativity constraints can be omitted. Finally, by substituting the Bethe approximation (29) into the variational free energy expression (28), the specific BFE expression can be obtained as   

(38) $$\begin{array}{cc} \; & F_{B}\left(b,q\right)=\sum_{r=1}^{R}\left\{D\left[b_{Y,r}∥\;f_{Y,r}\right]+D\left[b_{Z,r}∥\;f_{Z,r}\right]+H\left[q_{Z,r}\right]\right\}+\sum_{j=1}^{J}\left\{D\left[b_{H,j}∥\;f_{H,j}\right]+RH\left[q_{H,j}\right]\right\}\\ \; & +\sum_{k=1}^{K}\left\{D\left[b_{A,k}∥\;f_{A,k}\right]+DLH\left[q_{A,k}\right]\right\}+\sum_{j=1}^{J}\left\{D\left[b_{S,j}∥\;f_{S,j}\right]+\left(2-\delta \left[\;\mathrm{mod}\;\left(j/D\right)\right]\right)H\left[q_{S,j}\right]\right\}\\ \; & +\sum_{j=1}^{J}\left\{D\left[b_{G,j}∥\;f_{G,j}\right]+H\left[q_{G,j}\right]+D\left[b_{\Omega ,j}∥\;f_{\Omega ,j}\right]+\left|N_{j}\right|H\left[q_{\Omega ,j}\right]\right\}. \end{array}$$

Thus, the original BFE minimization problem can be formulated as

(39) $$\begin{array}{c} \min_{b,q}F_{B}\left(b,q\right),\;s.t.\;\;(30)–(37).\; \end{array}$$

By solving the problem (39), the optimal solutions for the factor beliefs and variable beliefs can be obtained. Using the optimal variable belief $q_{H,j}^{⋆}\left(h_{j}\right)$, the marginal posterior probability density $p\left(h_{j}|y;G\right)$ can be well approximated.

### 4.2. Constraint Reconstruction

The Bethe approximation introduces auxiliary beliefs through factor relationships in the joint posterior probability density, aiming to maintain the connections between probability factors and to avoid the complex integrals involved in computing the marginal posterior probability densities. However, since the original marginal consistency constraints still contain some high-dimensional integrals, these constraints need to be reconstructed into a more manageable form to reduce the computational burden.

To simplify the factor belief $b_{G,j}\left(g_{j},\omega_{j}\right)$, the latent precision $\omega_{j}$ can be assumed to be independent of other variables, which allows it to be separated from $b_{G,j}\left(g_{j},\omega_{j}\right)$ through a factorization constraint as

(40) $$\begin{array}{c} b_{G,j}\left(g_{j},\omega_{j}\right)=b_{\tilde{G},j}\left(g_{j}\right)\prod_{j^{\prime }\in N_{j}}q_{\Omega ,j}\left(\omega_{j^{\prime }}\right), \end{array}$$

where $b_{\tilde{G},j}\left(g_{j}\right)$ is the factor belief after separation. It can be observed that $b_{\tilde{G},j}\left(g_{j}\right)$ still satisfies the normalization constraint, which is given by

(41) $$\begin{array}{c} E_{b_{\tilde{G},j}}\left[1\right]=\int b_{\tilde{G},j}\left(g_{j}\right)dg_{j}=1. \end{array}$$

Further, by substituting the factorization constraint (40) into the KL divergence $D\left[b_{G,j}∥\;f_{G,j}\right]$, we obtain

(42) $$\begin{array}{cc} \; & D\left[b_{G,j}∥\;f_{G,j}\right]=\int b_{\tilde{G},j}\left(g_{j}\right)\prod_{j^{\prime }\in N_{j}}q_{\Omega ,j^{\prime }}\left(\omega_{j^{\prime }}\right)\ln\frac{b_{\tilde{G},j}\left(g_{j}\right)\prod_{j^{\prime }\in N_{j}}q_{\Omega ,j^{\prime }}\left(\omega_{j^{\prime }}\right)}{f_{G,j}\left(g_{j},\omega_{j}\right)}dg_{j}d\omega_{j}\\ \; & =-\int b_{\tilde{G},j}\left(g_{j}\right)\prod_{j^{\prime }\in N_{j}}q_{\Omega ,j^{\prime }}\left(\omega_{j^{\prime }}\right)\ln f_{G,j}\left(g_{j},\omega_{j}\right)dg_{j}d\omega_{j}-H\left[b_{\tilde{G},j}\right]-\sum_{j^{\prime }\in N_{j}}H\left[q_{\Omega ,j^{\prime }}\right]. \end{array}$$

By substituting the marginal consistency constraint (34) and the factorization constraint (40) back into the BFE $F_{B}\left(b,q\right)$ in (38), the channel value part in the last line of (38) can be rewritten as

(43) $$\begin{array}{cc} F_{\mathrm{cv}}\left(b,q\right) & =\sum_{j=1}^{J}\left\{D\left[b_{G,j}∥\;f_{G,j}\right]+H\left[q_{G,j}\right]+D\left[b_{\Omega ,j}∥\;f_{\Omega ,j}\right]+\left|N_{j}\right|H\left[q_{\Omega ,j}\right]\right\}\\ \; & =\sum_{j=1}^{J}\left\{H\left[q_{G,j}\right]+D\left[b_{\Omega ,j}∥\;f_{\Omega ,j}\right]-H\left[b_{\tilde{G},j}\right]\right\}-\sum_{j=1}^{J}\int b_{\tilde{G},j}\left(g_{j}\right)\prod_{j^{\prime }\in N_{j}}b_{\Omega ,j^{\prime }}\left(\omega_{j^{\prime }}\right)\ln f_{G,j}\left(g_{j},\omega_{j}\right)dg_{j}d\omega_{j}, \end{array}$$

Then, by replacing the BFE $F_{B}\left(b,q\right)$ in (38) with the expression in (43), it is denoted as $F_{\tilde{B}}\left(b,q\right)$. For further simplification, following the idea of expectation propagation approximation, the marginal consistency constraint (30) can be relaxed to the following mean and variance consistency constraints:

(44) $$\begin{array}{cc} \; & E_{q_{Z,r}}\left[z_{r}\right]=E_{b_{Y,r}}\left[z_{r}\right]=E_{b_{Z,r}}\left[z_{r}\right], \end{array}$$

(45) $$\begin{array}{cc} \; & {\mathrm{Var}}_{q_{Z,r}}\left[z_{r}\right]={\mathrm{Var}}_{b_{Y,r}}\left[z_{r}\right]={\mathrm{Var}}_{b_{Z,r}}\left[z_{r}\right]. \end{array}$$

For the constraint (31), considering that the factor belief $b_{Z,r}\left(z_{r},h\right)$ under any *r* includes the variable $h_{j}$, an average variance consistency constraint can be used to further reduce the complexity. To ensure estimation accuracy, the mean consistency constraint remains unchanged, yielding   

(46) $$\begin{array}{cc} \; & E_{q_{H,j}}\left[h_{j}\right]=E_{b_{Z,r}}\left[h_{j}\right]=E_{b_{H,j}}\left[h_{j}\right], \end{array}$$

(47) $$\begin{array}{cc} \; & {\mathrm{Var}}_{q_{H,j}}\left[h_{j}\right]=\frac{1}{R}\sum_{r=1}^{R}{\mathrm{Var}}_{b_{Z,r}}\left[h_{j}\right]={\mathrm{Var}}_{b_{H,j}}\left[h_{j}\right], \end{array}$$

Similarly, since $g_{j}$ is a continuous variable, the constraint (33) can be relaxed by applying mean and variance consistency constraints as

(48) $$\begin{array}{cc} \; & E_{q_{G,j}}\left[g_{j}\right]=E_{b_{H,j}}\left[g_{j}\right]=E_{b_{\tilde{G},j}}\left[g_{j}\right], \end{array}$$

(49) $$\begin{array}{cc} \; & {\mathrm{Var}}_{q_{G,j}}\left[g_{j}\right]={\mathrm{Var}}_{b_{H,j}}\left[g_{j}\right]={\mathrm{Var}}_{b_{\tilde{G},j}}\left[g_{j}\right]. \end{array}$$

Since the integration of binary variables is relatively simple, there is no need to relax the constraints (32) and (35). Combining the reconstruction design above, the BFE minimization problem under hybrid constraints can be finally formulated as

(50) $$\begin{array}{cc} \; & \min_{b,q,G}F_{\tilde{B}}\left(b,q;G\right),\;s.t.\\ \; & \;\mathrm{marginal}\;\mathrm{consistency}:\;(32),\;(35),\\ \; & \;\mathrm{normalization}:\;(36),\;(41),\\ \; & \;\mathrm{mean}\;\mathrm{and}\;\mathrm{variance}\;\mathrm{consistency}:\;\;(44)–(49). \end{array}$$

where G is treated as an unknown model parameter and is optimized together with the factor beliefs and variable beliefs in problem (50).

## 5. Theoretical Foundation of Correlated Hybrid Message Passing

This section aims to solve the aforementioned constrained BFE minimization problem to develop the hybrid message passing algorithm for joint active user detection and channel estimation. In the algorithm design, Lagrange multipliers are first used to weight the constraints, constructing the Lagrangian function. Then, the first-order partial derivatives of the Lagrangian function with respect to the beliefs, model parameters, and Lagrange multipliers are set to zero, yielding a set of stationary point equations. These equations include expressions for the beliefs, model parameters, and hybrid constraints. Finally, by solving the stationary point equations, update rules for the Lagrange multipliers or auxiliary variables can be obtained.

### 5.1. Lagrangian Function Construction

This subsection introduces the corresponding Lagrange multipliers for the hybrid constraints in problem (50) and constructs the required Lagrangian function accordingly. To solve the constrained minimization problem, we construct the Lagrangian function by introducing Lagrange multipliers for each of the hybrid constraints. The complete Lagrangian function can be decomposed into the following six parts:

(51) $$\begin{array}{c} L_{B}=F_{\tilde{B}}+L_{Z}+L_{H}+L_{A}+L_{S}+L_{G}, \end{array}$$

where $F_{\tilde{B}}$ represents the BFE, and $L_{Z}$, $L_{H}$, $L_{A}$, $L_{S}$, and $L_{G}$ denote the Lagrangian function components corresponding to the five types of beliefs in (29). Specifically, $L_{Z}$ corresponds to the signal transmission-type beliefs $b_{Y,r}\left(z_{r}\right)$, $q_{Z,r}\left(z_{r}\right)$, and $b_{Z,r}\left(z_{r}\right)$ with respect to the constraints (44), (45) and the relevant parts of constraint (36), expressed as   

(52) LZ=∑r=1R2Reτrz,bY*EbY,rzr−EqZ,rzr+ηrz,bYVarqZ,rzr−VarbY,rzr+∑r=1R2Reτrz,bZ*EbZ,rzr−EqZ,rzr+ηrz,bZVarqZ,rzr−VarbZ,rzr+∑r=1RκrqZEqZ,r1−1+κrbYEbY,r1−1+κrbZEbZ,r1−1.

Here, the symbol τ represents the Lagrange multipliers for the mean consistency constraints, η represents the Lagrange multipliers for the variance consistency constraints, and κ represents the Lagrange multipliers for the normalization constraints. Then, $L_{H}$ corresponds to the constraints on the equivalent channel-type beliefs $q_{H,j}\left(h_{j}\right)$ and $b_{H,j}\left(h_{j},\alpha_{k},s_{j},g_{j}\right)$ in the relevant parts of constraints (46), (47), and (36), which is given by

(53) LH=∑j=1J2Re(τr,jh,bZ)*EbZ,rhj−EqH,jhj+∑j=1Jηjh,bYRVarqH,jhj−∑r=1RVarbZ,rhj+∑j=1J2Re(τjh,bH)*EbH,jhj−EqH,jhj+∑j=1Jηjh,bHVarqH,jhj−VarbH,jhj+∑j=1JκjqHEqH,j1−1+κjbHEbH,j1−1.

Next, $L_{A}$ corresponds to the constraints on the activity indicator-type beliefs $q_{A,k}\left(\alpha_{k}\right)$ and $b_{A,k}\left(\alpha_{k}\right)$ in the relevant parts of constraints (32) and (36), expressed as

(54) $$\begin{array}{cc} L_{A} & =\sum_{j=1}^{J}\sum_{\alpha_{k}}\mu_{j}^{\alpha ,b_{H}}\left(\alpha_{k}\right)\left(q_{A,k}\left(\alpha_{k}\right)-\int \sum_{s_{j}}b_{H,j}\left(h_{j},\alpha_{k},s_{j},g_{j}\right)dh_{j}dg_{j}\right)\\ \; & +\sum_{k=1}^{K}\sum_{\alpha_{k}}\mu_{k}^{\alpha ,b_{A}}\left(\alpha_{k}\right)\left(q_{A,k}\left(\alpha_{k}\right)-b_{A,k}\left(\alpha_{k}\right)\right)+\sum_{k=1}^{K}\left\{\kappa_{k}^{q_{A}}\left(E_{q_{A,k}}\left[1\right]-1\right)+\kappa_{k}^{b_{A}}\left(E_{b_{A,k}}\left[1\right]-1\right)\right\}, \end{array}$$

where the symbol μ represents the Lagrange multipliers for the marginal consistency constraints. Further, $L_{S}$ corresponds to the constraints on the channel support beliefs $q_{S,d,l,k}\left(s_{d,l,k}\right)$ and $b_{S,d,l,k}\left(s_{d,l,k},s_{d-1,l,k}\right)$ in the relevant parts of constraints (35) and (36), which can be specifically written as   

(55) $$\begin{array}{cc} L_{S} & =\sum_{d,l,k}\sum_{s_{d,l,k}}\mu_{d,l,k}^{s,b_{H}}\left(s_{d,l,k}\right)\left(q_{S,d,l,k}\left(s_{d,l,k}\right)-\int \sum_{\alpha_{k}}b_{H,d,l,k}dh_{d,l,k}dg_{d,l,k}\right)\\ \; & +\sum_{l,k}\sum_{s_{1,l,k}}\mu_{1,l,k}^{s,b_{S}}\left(s_{1,l,k}\right)\left(q_{S,1,l,k}\left(s_{1,l,k}\right)-b_{S,1,l,k}\left(s_{1,l,k}\right)\right)\\ \; & +\sum_{l,k}\sum_{d=2}^{D}\sum_{s_{d,l,k}}\mu_{d,l,k}^{s,b_{S}}\left(s_{d,l,k}\right)\left(q_{S,d,l,k}\left(s_{d,l,k}\right)-\sum_{s_{d-1,l,k}}b_{S,d,l,k}\left(s_{d,l,k},s_{d-1,l,k}\right)\right)\\ \; & +\sum_{l,k}\sum_{d=1}^{D-1}\sum_{s_{d,l,k}}\mu_{d,l,k}^{s,b_{S}+}\left(s_{d,l,k}\right)\left(q_{S,d,l,k}\left(s_{d,l,k}\right)-\sum_{s_{d+1,l,k}}b_{S,d+1,l,k}\left(s_{d+1,l,k},s_{d,l,k}\right)\right)\\ \; & +\sum_{d,l,k}\left\{\kappa_{d,l,k}^{q_{S}}\left(E_{q_{S,d,l,k}}\left[1\right]-1\right)+\kappa_{d,l,k}^{b_{S}}\left(E_{b_{S,d,l,k}}\left[1\right]-1\right)\right\}. \end{array}$$

Finally, $L_{G}$ corresponds to the constraints on the channel value-type beliefs $q_{G,j}\left(g_{j}\right)$, $b_{\tilde{G},j}\left(g_{j}\right)$, and $q_{\Omega ,j}\left(\omega_{j}\right)$ in the relevant parts of constraints (41), (46), (47), and (36), given in   

(56) LG=∑j=1J2Reτjg,bH*EqG,jgj−EbH,jgj+ηjg,bHVarbH,jgj−VarqG,jgj=∑j=1J2Reτjg,bG˜*EqG,jgj−EbG˜,jgj+ηjg,bG˜VarbG˜,jgj−VarqG,jgj+∑j=1JκjqGEqG,j1−1+κjbG˜EbG˜,j1−1+κjbΩEqΩ,j1−1.

### 5.2. Belief Representation

For the Lagrangian function (51), by setting the first-order partial derivatives with respect to the auxiliary beliefs and model parameters to zero and solving, we derive the expressions for the factor beliefs, variable beliefs, and model parameters. By combining these expressions with the hybrid constraints, the stationary point equations can be formulated. These equations can be solved according to the modular structure of the factor graph in Figure 2, leading to the hybrid message passing algorithm. Due to the connections between probabilities, some beliefs are shared among modules. For ease of reference, we next list the specific representations of the factor beliefs and variable beliefs.

First, the specific expressions for the signal transmission-type beliefs $b_{Y,r}\left(z_{r}\right)$, $q_{Z,r}\left(z_{r}\right)$, and $b_{Z,r}\left(z_{r}\right)$ are provided. The factor belief $b_{Y,r}\left(z_{r}\right)$ is given by

(57) $$\begin{array}{c} b_{Y,r}\left(z_{r}\right)\propto f_{Y,r}\left(z_{r}\right)\mathrm{CN}\left(z_{r};\underset{\overset{\Delta }{\;=}\xi_{r}^{z,b_{Y}}}{\underset{︸}{E_{b_{Y,r}}\left[z_{r}\right]+\frac{\tau_{r}^{z,b_{Y}}}{\eta_{r}^{z,b_{Y}}}}},\frac{1}{\eta_{r}^{z,b_{Y}}}\right), \end{array}$$

and the variable belief $q_{Z,r}\left(z_{r}\right)$ is expressed as

(58) $$\begin{array}{c} q_{Z,r}\left(z_{r}\right)=\mathrm{CN}\left(z_{r};E_{q_{Z,r}}\left[z_{r}\right]+\frac{\tau_{r}^{z,b_{Y}}+\tau_{r}^{z,b_{Z}}}{\eta_{r}^{z,b_{Y}}+\eta_{r}^{z,b_{Z}}},\frac{1}{\eta_{r}^{z,b_{Y}}+\eta_{r}^{z,b_{Z}}}\right), \end{array}$$

while the factor belief $b_{Z,r}\left(z_{r},h\right)$ is expressed as

(59) $$\begin{array}{cc} b_{Z,r}\left(z_{r},h\right) & \propto f_{Z,r}\left(z_{r},h\right)\mathrm{CN}\left(z_{r};\underset{\overset{\Delta }{\;=}\xi_{r}^{z,b_{Z}}}{\underset{︸}{E_{b_{Z,r}}\left[z_{r}\right]+\frac{\tau_{r}^{z,b_{Z}}}{\eta_{r}^{z,b_{Z}}}}},\frac{1}{\eta_{r}^{z,b_{Z}}}\right)\mathrm{CN}\left(h_{j};\underset{\overset{\Delta }{\;=}\xi_{r,j}^{h,b_{Z}}}{\underset{︸}{E_{b_{Z,r}}\left[h_{j}\right]+\frac{\tau_{r,j}^{h,b_{Z}}}{\eta_{j}^{h,b_{Z}}}}},\frac{1}{\eta_{j}^{h,b_{Z}}}\right). \end{array}$$

Next, the specific expressions for the equivalent channel-type beliefs $q_{H,j}\left(h_{j}\right)$ and $b_{H,j}\left(h_{j},\alpha_{k},s_{j},g_{j}\right)$ are shown below. The variable belief $q_{H,j}\left(h_{j}\right)$ is given by

(60) $$\begin{array}{c} q_{H,j}\left(h_{j}\right)=\mathrm{CN}\left(h_{j};E_{q_{H,j}}\left[h_{j}\right]+\frac{\sum_{r=1}^{R}\tau_{r,j}^{h,b_{Z}}+\tau_{j}^{h,b_{H}}}{R\eta_{j}^{h,b_{Z}}+\eta_{j}^{h,b_{H}}},\frac{R}{R\eta_{j}^{h,b_{Z}}+\eta_{j}^{h,b_{H}}}\right), \end{array}$$

and the factor belief $b_{H,j}\left(h_{j},\alpha_{k},s_{j},g_{j}\right)$ is given by

(61) $$\begin{array}{cc} b_{H,j}\left(h_{j},\alpha_{k},s_{j},g_{j}\right) & \propto f_{H,j}\left(h_{j},\alpha_{k},s_{j},g_{j}\right)\mathrm{CN}\left(h_{j};\underset{\overset{\Delta }{\;=}\xi_{j}^{h,b_{H}}}{\underset{︸}{E_{b_{H,j}}\left[h_{j}\right]+\frac{\tau_{j}^{h,b_{H}}}{\eta_{j}^{h,b_{H}}}}},\frac{1}{\eta_{j}^{h,b_{H}}}\right)\\ \; & \times \exp\left(\mu_{j}^{\alpha ,b_{H}}\left(\alpha_{k}\right)+\mu_{j}^{s,b_{H}}\left(s_{j}\right)\right)\mathrm{CN}\left(g_{j};\underset{\overset{\Delta }{\;=}\xi_{j}^{g,b_{H}}}{\underset{︸}{E_{b_{H,j}}\left[g_{j}\right]+\frac{\tau_{j}^{g,b_{H}}}{\eta_{j}^{g,b_{H}}}}},\frac{1}{\eta_{j}^{g,b_{H}}}\right). \end{array}$$

Then, we present the specific expressions for the activity indicator beliefs $q_{A,k}\left(\alpha_{k}\right)$ and $b_{A,k}\left(\alpha_{k}\right)$. The variable belief $q_{A,k}\left(\alpha_{k}\right)$ is given by

(62) $$\begin{array}{c} q_{A,k}\left(\alpha_{k}\right)\propto \exp\left(\frac{1}{DL}\left(\mu_{k}^{\alpha ,b_{A}}\left(\alpha_{k}\right)+\sum_{d,l}\mu_{d,l,k}^{\alpha ,b_{H}}\left(\alpha_{k}\right)\right)\right), \end{array}$$

and the factor belief $b_{A,k}\left(\alpha_{k}\right)$ is expressed as

(63) $$\begin{array}{c} b_{A,k}\left(\alpha_{k}\right)\propto f_{A,k}\left(\alpha_{k}\right)\exp\left(\mu_{k}^{\alpha ,b_{A}}\left(\alpha_{k}\right)\right). \end{array}$$

Furthermore, the specific expressions for the channel support-type beliefs $q_{S,d,l,k}\left(s_{d,l,k}\right)$ and $b_{S,d,l,k}\left(s_{d,l,k},s_{d-1,l,k}\right)$ are provided. The variable belief $q_{S,d,l,k}\left(s_{d,l,k}\right)$ is given by

(64) $$\begin{array}{c} q_{S,d,l,k}\left(s_{d,l,k}\right)\propto \left\{\begin{array}{c} \exp\left(\frac{1}{2}\left(\mu_{d,l,k}^{s,b_{H}}\left(s_{d,l,k}\right)+\mu_{d,l,k}^{s,b_{S}}\left(s_{d,l,k}\right)+\mu_{d,l,k}^{s,b_{S}+}\left(s_{d,l,k}\right)\right)\right),\\ \;\;\;\;\;\;\;\;\;\;d=1,\cdots ,D-1,\\ \exp\left(\mu_{d,l,k}^{s,b_{H}}\left(s_{d,l,k}\right)+\mu_{d,l,k}^{s,b_{S}}\left(s_{d,l,k}\right)\right),\;d=D. \end{array}\right. \end{array}$$

When d=2,⋯,D, the factor belief $b_{S,d,l,k}\left(s_{d,l,k},s_{d-1,l,k}\right)$ is written as

(65) $$\begin{array}{c} b_{S,d,l,k}\left(s_{d,l,k},s_{d-1,l,k}\right)\propto f_{S,d,l,k}\left(s_{d,l,k},s_{d-1,l,k}\right)\exp\left(\mu_{d,l,k}^{s,b_{S}}\left(s_{d,l,k}\right)+\mu_{d-1,l,k}^{s,b_{S}+}\left(s_{d-1,l,k}\right)\right). \end{array}$$

When d=1, the factor belief $b_{S,1,l,k}\left(s_{1,l,k}\right)$ is given by

(66) $$\begin{array}{c} b_{S,1,l,k}\left(s_{1,l,k}\right)\propto f_{S,1,l,k}\left(s_{1,l,k}\right)\exp\left(\mu_{1,l,k}^{s,b_{S}}\left(s_{1,l,k}\right)\right). \end{array}$$

Finally, the specific expressions for the channel value beliefs $q_{G,j}\left(g_{j}\right)$, $b_{G,j}^{\prime }\left(g_{j}\right)$, and $b_{\Omega ,j}\left(\omega_{j}\right)$ are given. The variable belief $q_{G,j}\left(g_{j}\right)$ is expressed as

(67) $$\begin{array}{c} q_{G,j}\left(g_{j}\right)=\mathrm{CN}\left(g_{j};E_{q_{G,j}}\left[g_{j}\right]+\frac{\tau_{j}^{g,b_{H}}+\tau_{j}^{g,b_{\tilde{G}}}}{\eta_{j}^{g,b_{H}}+\eta_{j}^{g,b_{\tilde{G}}}},\frac{1}{\eta_{j}^{g,b_{H}}+\eta_{j}^{g,b_{\tilde{G}}}}\right), \end{array}$$

the factor belief $b_{\tilde{G},j}\left(g_{j}\right)$ is given by   

(68) $$\begin{array}{c} b_{\tilde{G},j}\left(g_{j}\right)\propto \mathrm{CN}\left(g_{j};\underset{\overset{\Delta }{\;=}\xi_{j}^{g,b_{\tilde{G}}}}{\underset{︸}{E_{b_{\tilde{G},j}}\left[g_{j}\right]+\frac{\tau_{j}^{g,b_{\tilde{G}}}}{\eta_{j}^{g,b_{\tilde{G}}}}}},\frac{1}{\eta_{j}^{g,b_{\tilde{G}}}}\right)\exp\left(\int \prod_{j^{\prime }\in N_{j}}b_{\Omega ,j^{\prime }}\left(\omega_{j^{\prime }}\right)\ln f_{G,j}\left(g_{j},\omega_{j}\right)d\omega_{j}\right), \end{array}$$

and the factor belief $b_{\Omega ,j}\left(\omega_{j}\right)$ is given by   

(69) $$\begin{array}{c} b_{\Omega ,j}\left(\omega_{j}\right)\propto f_{\Omega ,j}\left(\omega_{j}\right)\prod_{j^{\prime }\in N_{j}}\exp\left(\int b_{\tilde{G},j^{\prime }}\left(g_{j^{\prime }}\right)\prod_{j^{\prime \prime }\in N_{j^{\prime }\setminus j}}b_{\Omega ,j^{\prime \prime }}\left(\omega_{j^{\prime \prime }}\right)\ln f_{G,j^{\prime }}\left(g_{j^{\prime }},\omega_{j^{\prime }}\right)dg_{j^{\prime }}d\omega_{j^{\prime }\setminus j}\right). \end{array}$$

### 5.3. Signal Transmission Module

This subsection will detail the message passing process of the signal transmission module. By simplifying the mean expression of the factor belief $b_{Y,r}\left(z_{r}\right)$ in (57), we can obtain

(70) $$\begin{array}{c} E_{b_{Y,r}}\left[z_{r}\right]=y_{r}+\sigma \tau_{r}^{z,b_{Y}}. \end{array}$$

Based on the mean expression of the variable belief $q_{Z,r}\left(z_{r}\right)$ in (58), we can obtain $\tau_{r}^{z,b_{Y}}=-\tau_{r}^{z,b_{Z}}$. By substituting the factor belief $b_{Y,r}\left(z_{r}\right)$ from (57) and the variable belief $q_{Z,r}\left(z_{r}\right)$ from (58) into the variance consistency constraint ${\mathrm{Var}}_{q_{Z,r}}\left[z_{r}\right]={\mathrm{Var}}_{b_{Y,r}}\left[z_{r}\right]$ in (45), we get $\eta_{r}^{z,b_{Z}}=1/\sigma$. Combining the equations $\tau_{r}^{z,b_{Y}}=-\tau_{r}^{z,b_{Z}}$, $\eta_{r}^{z,b_{Z}}=1/\sigma$, and (70), the auxiliary mean $\xi_{r}^{z,b_{Z}}$ in (59) can be rewritten as

(71) $$\begin{array}{c} \xi_{r}^{z,b_{Z}}=-\tau_{r}^{z,b_{Y}}\sigma +E_{b_{Y,r}}\left[z_{r}\right]=y_{r}. \end{array}$$

Then, based on the variance expression of the variable belief $q_{H,j}\left(h_{j}\right)$ in (60), the variance consistency constraint ${\mathrm{Var}}_{q_{H,j}}\left[h_{j}\right]={\mathrm{Var}}_{b_{H,j}}\left[h_{j}\right]$ in (47) can be re-expressed as

(72) $$\begin{array}{c} \eta_{j}^{h,b_{Z}}=1/{\mathrm{Var}}_{b_{H,j}}\left[h_{j}\right]-\frac{\eta_{j}^{h,b_{H}}}{R}. \end{array}$$

By substituting the variable belief $q_{Z,r}\left(z_{r}\right)$ from (58) and the factor belief $b_{Z,r}\left(z_{r},h\right)$ from (59) into the variance consistency constraint ${\mathrm{Var}}_{q_{Z,r}}\left[z_{r}\right]={\mathrm{Var}}_{b_{Z,r}}\left[z_{r}\right]$ in (45), and further simplifying this constraint, we can obtain

(73) $$\begin{array}{c} 1/\eta_{r}^{z,b_{Y}}=\sum_{j=1}^{J}{\left|\varphi_{r,j}\right|}^{2}/\eta_{j}^{h,b_{Z}}. \end{array}$$

Next, based on the mean consistency constraints (44) and (46), the auxiliary mean $\xi_{r}^{z,b_{Y}}$ in (57) can be given by

(74) $$\begin{array}{c} \xi_{r}^{z,b_{Y}}=\frac{\tau_{r}^{z,b_{Y}}}{\eta_{r}^{z,b_{Y}}}+\sum_{j=1}^{J}\varphi_{r,j}E_{b_{H,j}}\left[h_{j}\right]. \end{array}$$

According to the definitions of the auxiliary mean $\xi_{r}^{z,b_{Y}}$ in Equation (57) and the auxiliary mean $\xi_{r}^{z,b_{Z}}$ in (59), we have

(75) $$\begin{array}{c} \tau_{r}^{z,b_{Y}}=\frac{\xi_{r}^{z,b_{Y}}-\xi_{r}^{z,b_{Z}}}{1/\eta_{r}^{z,b_{Y}}+1/\eta_{r}^{z,b_{Z}}}=\frac{\xi_{r}^{z,b_{Y}}-y_{r}}{1/\eta_{r}^{z,b_{Y}}+\sigma }. \end{array}$$

Then, based on the factor belief $b_{Z,r}\left(z_{r},h\right)$ in Equation (59), we calculate the mean expressions $E_{b_{Y,r}}\left[z_{r}\right]$ and $E_{b_{Y,r}}\left[h_{j}\right]$. By connecting and simplifying these means, the relationship between the Lagrange multipliers $\tau_{r,j}^{h,b_{Z}}$ and $\tau_{r}^{z,b_{Y}}$ can be derived as follows:

(76) $$\begin{array}{c} \tau_{r,j}^{h,b_{Z}}=-\varphi_{r,j}^{*}\tau_{r}^{z,b_{Z}}=\varphi_{r,j}^{*}\tau_{r}^{z,b_{Y}}, \end{array}$$

Furthermore, by substituting the factor belief $b_{Z,r}\left(z_{r},h\right)$ from (59) and the variable belief $q_{H,j}\left(h_{j}\right)$ from (60) into the variance consistency constraint ${\mathrm{Var}}_{q_{H,j}}\left[h_{j}\right]=\frac{1}{R}\sum_{r=1}^{R}{\mathrm{Var}}_{b_{Z,r}}\left[h_{j}\right]$ in (47) and simplifying, we derive

(77) $$\begin{array}{c} 1/\eta_{j}^{h,b_{H}}=1/\left(\sum_{r=1}^{R}\frac{{\left|\varphi_{r,j}\right|}^{2}}{1/\eta_{r}^{z,b_{Y}}+\sigma }\right)-\frac{1}{R\eta_{j}^{h,b_{Z}}}, \end{array}$$

Finally, based on the mean expression of the variable belief $q_{H,j}\left(h_{j}\right)$ in Equation (60), we obtain $\tau_{j}^{h,b_{H}}=-\sum_{r=1}^{R}\tau_{r,j}^{h,b_{Z}}$. Combining this with (76), the auxiliary mean $\xi_{j}^{h,b_{H}}$ in (61) can be reconstructed as

(78) $$\begin{array}{c} \xi_{j}^{h,b_{H}}=E_{b_{H,j}}\left[h_{j}\right]-\frac{1}{\eta_{j}^{h,b_{H}}}\sum_{r=1}^{R}\varphi_{r,j}^{*}\tau_{r}^{z,b_{Y}}. \end{array}$$

### 5.4. Equivalent Channel Module

This subsection will detail the message passing process of the equivalent channel module. Based on the factor belief $b_{H,j}\left(h_{j},\alpha_{k},s_{j},g_{j}\right)$ in (61), the  auxiliary messages are introduced as follows:

(79) $$\begin{array}{cc} \; & v_{j}^{\alpha ,b_{H}}\left(\alpha_{k}\right)\propto \exp\left(\mu_{j}^{\alpha ,b_{H}}\left(\alpha_{k}\right)\right)\overset{\Delta }{\;=}\frac{1}{1+\exp\left(-\gamma_{j}^{\alpha ,b_{H}}\right)}\delta \left[\alpha_{k}-1\right]+\frac{1}{1+\exp\left(\gamma_{j}^{\alpha ,b_{H}}\right)}\delta \left[\alpha_{k}\right], \end{array}$$

(80) $$\begin{array}{cc} \; & v_{j}^{s,b_{H}}\left(s_{j}\right)\propto \exp\left(\mu_{j}^{s,b_{H}}\left(s_{j}\right)\right)\overset{\Delta }{\;=}\frac{1}{1+\exp\left(-\gamma_{j}^{s,b_{H}}\right)}\delta \left[s_{j}-1\right]+\frac{1}{1+\exp\left(\gamma_{j}^{s,b_{H}}\right)}\delta \left[s_{j}\right], \end{array}$$

where $\gamma_{j}^{\alpha ,b_{H}}=\ln\frac{v_{j}^{\alpha ,b_{H}}\left(\alpha_{k}=1\right)}{v_{j}^{\alpha ,b_{H}}\left(\alpha_{k}=0\right)}$ and $\gamma_{j}^{s,b_{H}}=\ln\frac{v_{j}^{s,b_{H}}\left(s_{j}=1\right)}{v_{j}^{s,b_{H}}\left(s_{j}=0\right)}$ are the log-likelihood ratios of $v_{j}^{\alpha ,b_{H}}\left(\alpha_{k}\right)$ and $v_{j}^{s,b_{H}}\left(s_{j}\right)$, respectively. Here, the subscript ${\left(\cdot \right)}_{j}$ is equivalent to the subscript ${\left(\cdot \right)}_{d,l,k}$. With these definitions, the factor belief $b_{H,j}\left(h_{j},\alpha_{k},s_{j},g_{j}\right)$ in (61) can be reformulated as

(81) $$\begin{array}{cc} \; & b_{H,j}\left(h_{j},\alpha_{k},s_{j},g_{j}\right)\\ \; & \propto \delta \left(h_{j}-\alpha_{k}s_{j}g_{j}\right)CN\left(h_{j};\xi_{j}^{h,b_{H}},1/\eta_{j}^{h,b_{H}}\right)v_{j}^{\alpha ,b_{H}}\left(\alpha_{k}\right)v_{j}^{s,b_{H}}\left(s_{j}\right)CN\left(g_{j};\xi_{j}^{g,b_{H}},1/\eta_{j}^{g,b_{H}}\right). \end{array}$$

First, we present the derivation related to $h_{j}$ in the equivalent channel module. By integrating the factor belief $b_{H,j}\left(h_{j},\alpha_{k},s_{j},g_{j}\right)$ in (81) over all variables except $h_{j}$, this yields the following equation:

(82) $$\begin{array}{c} \int \sum_{\alpha_{k},s_{j}}b_{H,j}\left(h_{j},\alpha_{k},s_{j},g_{j}\right)dg_{j}={\tilde{\rho }}_{j}^{b_{H}}\mathrm{CN}\left(h_{j};{\tilde{\xi }}_{j}^{b_{H}},{\tilde{\lambda }}_{j}^{b_{H}}\right)+\left(1-{\tilde{\rho }}_{j}^{b_{H}}\right)\delta \left(h_{j}\right), \end{array}$$

where ${\tilde{\rho }}_{j}^{b_{H}}$, ${\tilde{\xi }}_{j}^{b_{H}}$, and ${\tilde{\lambda }}_{j}^{b_{H}}$ are expressed as   

(83) $$\begin{array}{cc} {\tilde{\rho }}_{j}^{b_{H}} & =1/\{1+\left(\exp\left(-\gamma_{j}^{\alpha ,b_{H}}\right)+\exp\left(-\gamma_{j}^{s,b_{H}}\right)+\exp\left(-\gamma_{j}^{\alpha ,b_{H}}-\gamma_{j}^{s,b_{H}}\right)\right)CN\left(0;\xi_{j}^{h,b_{H}},1/\eta_{j}^{h,b_{H}}\right)\\ & /CN\left(0;\xi_{j}^{h,b_{H}}-\xi_{j}^{g,b_{H}},1/\eta_{j}^{h,b_{H}}+1/\eta_{j}^{g,b_{H}}\right)\}, \end{array}$$

(84) $$\begin{array}{cc} \; & \;\;\;{\tilde{\xi }}_{j}^{b_{H}}=\frac{\xi_{j}^{h,b_{H}}\eta_{j}^{h,b_{H}}+\xi_{j}^{g,b_{H}}\eta_{j}^{g,b_{H}}}{\eta_{j}^{h,b_{H}}+\eta_{j}^{g,b_{H}}}, \end{array}$$

(85) $$\begin{array}{cc} \; & \;\;\;{\tilde{\lambda }}_{j}^{b_{H}}=1/\left(\eta_{j}^{h,b_{H}}+\eta_{j}^{g,b_{H}}\right). \end{array}$$

Thus, the mean $E_{b_{H,j}}\left[h_{j}\right]$ and the variance ${\mathrm{Var}}_{b_{H,j}}\left[h_{j}\right]$ can be calculated as

(86) $$\begin{array}{c} E_{b_{H,j}}\left[h_{j}\right]={\tilde{\rho }}_{j}^{b_{H}}{\tilde{\xi }}_{j}^{b_{H}}, \end{array}$$

(87) $$\begin{array}{c} {\mathrm{Var}}_{b_{H,j}}\left[h_{j}\right]={\tilde{\rho }}_{j}^{b_{H}}\left(1-{\tilde{\rho }}_{j}^{b_{H}}\right){\left|{\tilde{\xi }}_{j}^{b_{H}}\right|}^{2}+{\tilde{\rho }}_{j}^{b_{H}}{\tilde{\lambda }}_{j}^{b_{H}}. \end{array}$$

Then, we provide the derivation related to $\alpha_{k}$ in the equivalent channel module. Based on (62) for the variable belief $q_{A,k}\left(\alpha_{k}\right)$, the auxiliary message $v_{j}^{b_{H},\alpha }\left(\alpha_{k}\right)$ is defined as

(88) $$\begin{array}{cc} \; & v_{j}^{b_{H},\alpha }\left(\alpha_{k}\right)\\ \; & \overset{\Delta }{\;=}\frac{1}{1+\exp\left(-\gamma_{j}^{b_{H},\alpha }\right)}\delta \left[\alpha_{k}-1\right]+\frac{1}{1+\exp\left(\gamma_{j}^{b_{H},\alpha }\right)}\delta \left[\alpha_{k}\right]\\ \; & \propto \exp\left(\frac{1}{DL}\left(\mu_{k}^{\alpha ,b_{A}}\left(\alpha_{k}\right)+\sum_{d^{\prime },l^{\prime }}\mu_{d^{\prime },l^{\prime },k}^{\alpha ,b_{H}}\left(\alpha_{k}\right)\right)-\mu_{d,l,k}^{\alpha ,b_{H}}\left(\alpha_{k}\right)\right), \end{array}$$

where $\gamma_{j}^{b_{H},\alpha }=\ln\frac{v_{j}^{b_{H},\alpha }\left(\alpha_{k}=1\right)}{v_{j}^{b_{H},\alpha }\left(\alpha_{k}=0\right)}$ is the log-likelihood ratio of $v_{j}^{b_{H},\alpha }\left(\alpha_{k}\right)$. By substituting the variable belief $q_{A,k}\left(\alpha_{k}\right)$ in (62) and the factor belief $b_{H,j}\left(h_{j},\alpha_{k},s_{j},g_{j}\right)$ in (81) into the marginal consistency constraint in (32) and simplifying this constraint, we can obtain

(89) $$\begin{array}{cc} \; & \gamma_{j}^{b_{H},\alpha }=\ln(\frac{1}{1+\exp\left(\gamma_{j}^{s,b_{H}}\right)}\\ \; & +\frac{CN\left(0;\xi_{j}^{h,b_{H}}-\xi_{j}^{g,b_{H}},1/\eta_{j}^{h,b_{H}}+1/\eta_{j}^{g,b_{H}}\right)}{\left(1+\exp\left(-\gamma_{j}^{s,b_{H}}\right)\right)CN\left(0;\xi_{j}^{h,b_{H}},1/\eta_{j}^{h,b_{H}}\right)}). \end{array}$$

Next, the following derivation concerns the part related to the channel support $s_{j}$. Based on the variable belief $q_{S,j}\left(s_{j}\right)$ in (64), the auxiliary message $v_{j}^{b_{H},s}\left(s_{j}\right)$ is defined as

(90) $$\begin{array}{cc} \; & v_{j}^{b_{H},s}\left(s_{j}\right)\\ \; & \overset{\Delta }{\;=}\frac{1}{1+\exp\left(-\gamma_{j}^{b_{H},s}\right)}\delta \left[s_{j}-1\right]+\frac{1}{1+\exp\left(\gamma_{j}^{b_{H},s}\right)}\delta \left[s_{j}\right]\\ \; & \propto \left\{\begin{array}{c} \exp(\frac{1}{2}(-\mu_{d,l,k}^{s,b_{H}}\left(s_{d,l,k}\right)+\mu_{d,l,k}^{s,b_{S}}\left(s_{d,l,k}\right)\\ \;\;+\mu_{d,l,k}^{s,b_{S}+}\left(s_{d,l,k}\right))),\;\;d=1,\cdots ,D-1,\\ \exp\left(\mu_{d,l,k}^{s,b_{S}}\left(s_{d,l,k}\right)\right),\;\;d=D, \end{array}\right. \end{array}$$

where $\gamma_{j}^{b_{H},s}=\ln\frac{v_{j}^{b_{H},s}\left(s_{j}=1\right)}{v_{j}^{b_{H},s}\left(s_{j}=0\right)}$ represents the log-likelihood ratio of $v_{j}^{b_{H},s}\left(s_{j}\right)$. By substituting the variable belief $q_{S,j}\left(s_{j}\right)$ in (64) and the factor belief $b_{H,j}\left(h_{j},\alpha_{k},s_{j},g_{j}\right)$ in (81) into the marginal consistency constraint in (35) and simplifying this constraint, we have   

(91) $$\begin{array}{cc} \; & \gamma_{j}^{b_{H},s}=\ln(\frac{1}{1+\exp\left(\gamma_{j}^{\alpha ,b_{H}}\right)}\\ \; & +\frac{CN\left(0;\xi_{j}^{h,b_{H}}-\xi_{j}^{g,b_{H}},1/\eta_{j}^{h,b_{H}}+\xi_{j}^{g,b_{H}}\right)}{\left(1+\exp\left(-\gamma_{j}^{\alpha ,b_{H}}\right)\right)CN\left(0;\xi_{j}^{h,b_{H}},1/\eta_{j}^{h,b_{H}}\right)}). \end{array}$$

Finally, the derivation related to $g_{j}$ is presented. By marginalizing the factor belief $b_{H,j}\left(h_{j},\alpha_{k},s_{j},g_{j}\right)$ in (81) over all variables except $g_{j}$, we can obtain its marginal belief as

(92) $$\begin{array}{cc} \; & \int \sum_{\alpha_{k},s_{j}}b_{H,j}\left(h_{j},\alpha_{k},s_{j},g_{j}\right)dh_{j}={\tilde{\rho }}_{j}^{b_{H}}CN\left(h_{j};{\tilde{\xi }}_{j}^{b_{H}},{\tilde{\lambda }}_{j}^{b_{H}}\right)\\ \; & \;\;+\left(1-{\tilde{\rho }}_{j}^{b_{H}}\right)CN\left(h_{j};\xi_{j}^{g,b_{H}},1/\eta_{j}^{g,b_{H}}\right), \end{array}$$

where ${\tilde{\rho }}_{j}^{b_{H}}$, $\tilde{\xi }j^{b_{H}}$, and $\tilde{\lambda }j^{b_{H}}$ are given in (83), (84), and (85), respectively. Therefore, $E_{b_{H,j}}\left[{\left|g_{j}\right|}^{2}\right]$ can be calculated as

(93) $$\begin{array}{cc} E_{b_{H,j}}\left[{\left|g_{j}\right|}^{2}\right] & ={\tilde{\rho }}_{j}^{b_{H}}\left({\left|{\tilde{\xi }}_{j}^{b_{H}}\right|}^{2}+{\tilde{\lambda }}_{j}^{b_{H}}\right)\\ \; & +\left(1-{\tilde{\rho }}_{j}^{b_{H}}\right)\left({\left|\xi_{j}^{g,b_{H}}\right|}^{2}+1/\eta_{j}^{g,b_{H}}\right). \end{array}$$

### 5.5. Activity Indicator Module

This subsection will detail the message passing process of the activity indicator module. Based on the variable belief $b_{A,k}\left(\alpha_{k}\right)$ in (63), an auxiliary message $v_{k}^{\alpha ,b_{H}}\left(\alpha_{k}\right)$ is introduced, which is expressed as

(94) $$\begin{array}{cc} \; & v_{k}^{\alpha ,b_{A}}\left(\alpha_{k}\right)\propto \exp\left(\mu_{k}^{\alpha ,b_{A}}\left(\alpha_{k}\right)\right)\\ \; & \overset{\Delta }{\;=}\frac{1}{1+\exp\left(-\gamma_{k}^{\alpha ,b_{A}}\right)}\delta \left[\alpha_{k}-1\right]+\frac{1}{1+\exp\left(\gamma_{k}^{\alpha ,b_{A}}\right)}\delta \left[\alpha_{k}\right], \end{array}$$

where $\gamma_{k}^{\alpha ,b_{A}}=\ln\frac{v_{k}^{\alpha ,b_{A}}\left(\alpha_{k}=1\right)}{v_{k}^{\alpha ,b_{A}}\left(\alpha_{k}=0\right)}$ is the log-likelihood ratio of $v_{k}^{\alpha ,b_{A}}\left(\alpha_{k}\right)$. By substituting the factor belief $b_{H,j}\left(h_{j},\alpha_{k},s_{j},g_{j}\right)$ in (61) and the variable belief $q_{A,k}\left(\alpha_{k}\right)$ in (62) into the marginal consistency constraint in (32) and simplifying, and by combining the definitions of the auxiliary messages $v_{j}^{b_{H},\alpha }\left(\alpha_{k}\right)$ in (88) and $v_{k}^{\alpha ,b_{A}}\left(\alpha_{k}\right)$ in (94), the following result is obtained:

(95) $$\begin{array}{c} \gamma_{k}^{\alpha ,b_{A}}=\sum_{d=1}^{D}\sum_{l=1}^{L}\gamma_{d,l,k}^{b_{H},\alpha }. \end{array}$$

Then, by substituting the variable belief $q_{A,k}\left(\alpha_{k}\right)$ from (62) and the factor belief $b_{A,k}\left(\alpha_{k}\right)$ from (63) into the marginal consistency constraint in (32) and simplifying it, and then combining this with the definitions of the auxiliary messages $v_{j}^{\alpha ,b_{H}}\left(\alpha_{k}\right)$ in (79) and $v_{j}^{b_{H},\alpha }\left(\alpha_{k}\right)$ in (88), we can get

(96) $$\begin{array}{cc} \gamma_{d,l,k}^{\alpha ,b_{H}} & =\sum_{d^{\prime }=1}^{D}\sum_{l^{\prime }=1}^{L}\gamma_{d^{\prime },l^{\prime },k}^{b_{H},\alpha }-\gamma_{d,l,k}^{b_{H},\alpha }+\ln\frac{\rho_{k}}{1-\rho_{k}}\\ \; & =\gamma_{k}^{\alpha ,b_{A}}-\gamma_{d,l,k}^{b_{H},\alpha }+\gamma_{k}^{b_{A}}, \end{array}$$

where $\gamma_{k}^{b_{A}}=\ln\frac{\rho_{k}}{1-\rho_{k}}$ represents the log-likelihood ratio of the active probability $\rho_{k}$. Next, by taking the first partial derivative of the Lagrangian function (51) with respect to the active probability $\rho_{k}$ and setting it to zero, the update rule for the active probability $\rho_{k}$ can be derived as

(97) $$\begin{array}{cc} \rho_{k} & =E_{b_{A,k}}\left[\alpha_{k}\right]\\ \; & =\frac{\rho_{k}^{\left(\mathrm{old}\right)}\rho_{k}^{\alpha ,b_{A}}}{\rho_{k}^{\left(\mathrm{old}\right)}\rho_{k}^{\alpha ,b_{A}}+\left(1-\rho_{k}^{\left(\mathrm{old}\right)}\right)\left(1-\rho_{k}^{\alpha ,b_{A}}\right)}, \end{array}$$

where the mean $E_{b_{A,k}}\left[\alpha_{k}\right]$ can be calculated according to (63). The expression $\rho_{k}^{\alpha ,b_{A}}=1/\left(1+\exp\left(-\gamma_{k}^{\alpha ,b_{A}}\right)\right)$ represents the updated active probability, where $\rho_{k}^{\left(\mathrm{old}\right)}$ denotes the active probability before the update. To avoid confusion, the superscript ${\left(\cdot \right)}^{\left(\mathrm{old}\right)}$ is used for distinction throughout this paper. Finally, (97) can be rewritten in the following equivalent form of the log-likelihood ratio:

(98) $$\begin{array}{c} \gamma_{k}^{b_{A}}=\ln\frac{\rho_{k}}{1-\rho_{k}}=\gamma_{k}^{b_{A}\left(\mathrm{old}\right)}+\gamma_{k}^{\alpha ,b_{A}}. \end{array}$$

### 5.6. Channel Support Module

This subsection will provide a detailed description of the message passing process in the channel support module. For ease of notation, the subscript ${\left(\cdot \right)}_{j}$ will be rewritten as ${\left(\cdot \right)}_{d,l,k}$ in this subsection. Referring to the auxiliary message $v_{d,l,k}^{s,b_{H}}\left(s_{d,l,k}\right)$ in (80), and based on the factor belief $b_{S,d,l,k}\left(s_{d,l,k},s_{d-1,l,k}\right)$ in (65), the following auxiliary messages are introduced:

(99) $$\begin{array}{cc} \; & v_{d,l,k}^{s,b_{S}}\left(s_{d,l,k}\right)\\ \; & \overset{\Delta }{\;=}\frac{1}{1+\exp(-\gamma_{d,l,k}^{s,b_{S}})}\delta \left[s_{d,l,k}-1\right]+\frac{1}{1+\exp(\gamma_{d,l,k}^{s,b_{S}})}\delta \left[s_{d,l,k}\right]\\ \; & \propto \exp\left(\mu_{d,l,k}^{s,b_{S}}\left(s_{d,l,k}\right)\right),\;\;\;d=1,\cdots ,D, \end{array}$$

(100) $$\begin{array}{cc} \; & v_{d,l,k}^{s,b_{S}+}\left(s_{d,l,k}\right)\\ \; & \overset{\Delta }{\;=}\frac{1}{1+\exp(-\gamma_{d,l,k}^{s,b_{S}+})}\delta \left[s_{d,l,k}-1\right]+\frac{1}{1+\exp(\gamma_{d,l,k}^{s,b_{S}+})}\delta \left[s_{d,l,k}\right]\\ \; & \propto \exp\left(\mu_{d,l,k}^{s,b_{S}+}\left(s_{d,l,k}\right)\right),\;d=1,\cdots ,D-1, \end{array}$$

where $\gamma_{d,l,k}^{s,b_{S}}=\ln\frac{v_{d,l,k}^{s,b_{S}}\left(s_{d,l,k}=1\right)}{v_{d,l,k}^{s,b_{S}}\left(s_{d,l,k}=0\right)}$ and $\gamma_{d,l,k}^{s,b_{S}+}=\ln\frac{v_{d,l,k}^{s,b_{S}+}\left(s_{d,l,k}=1\right)}{v_{d,l,k}^{s,b_{S}+}\left(s_{d,l,k}=0\right)}$ are the log-likelihood ratios of $v_{d,l,k}^{s,b_{S}}\left(s_{d,l,k}\right)$ and $v_{d,l,k}^{s,b_{S}+}\left(s_{d,l,k}\right)$, respectively. Referring to (90), the auxiliary message $v_{d,l,k}^{b_{S},s}\left(s_{d,l,k}\right)$ is defined as

(101) $$\begin{array}{cc} \; & v_{d,l,k}^{b_{S},s}\left(s_{d,l,k}\right)\\ \; & \overset{\Delta }{\;=}\frac{1}{1+\exp(-\gamma_{d,l,k}^{b_{S},s})}\delta \left[s_{d,l,k}-1\right]+\frac{1}{1+\exp(\gamma_{d,l,k}^{b_{S},s})}\delta \left[s_{d,l,k}\right]\\ \; & \propto \left\{\begin{array}{c} \exp\left(\frac{1}{2}\left(\mu_{d,l,k}^{s,b_{H}}\left(s_{d,l,k}\right)-\mu_{d,l,k}^{s,b_{S}}\left(s_{d,l,k}\right)+\mu_{d,l,k}^{s,b_{S}+}\left(s_{d,l,k}\right)\right)\right),\\ \;\;\;\;\;\;\;d=1,\cdots ,D-1,\\ \exp\left(\mu_{d,l,k}^{s,b_{H}}\left(s_{d,l,k}\right)\right),\;d=D, \end{array}\right. \end{array}$$

where $\gamma_{d,l,k}^{b_{S},s}=\ln\frac{v_{d,l,k}^{b_{S},s}\left(s_{d,l,k}=1\right)}{v_{d,l,k}^{b_{S},s}\left(s_{d,l,k}=0\right)}$ is the log-likelihood ratio of $v_{d,l,k}^{b_{S},s}\left(s_{d,l,k}\right)$. Similarly, define the auxiliary message $v_{d,l,k}^{b_{S}+,s}\left(s_{d,l,k}\right)$ as   

(102) $$\begin{array}{cc} \; & v_{d,l,k}^{b_{S}+,s}\left(s_{d,l,k}\right)\;\;\;d=1,\cdots ,D-1,\\ \; & \overset{\Delta }{\;=}\frac{1}{1+\exp(-\gamma_{d,l,k}^{b_{S}+,s})}\delta \left[s_{d,l,k}-1\right]+\frac{1}{1+\exp(\gamma_{d,l,k}^{b_{S}+,s})}\delta \left[s_{d,l,k}\right]\\ \; & \propto \exp\left(\frac{1}{2}\left(\mu_{d,l,k}^{s,b_{H}}\left(s_{d,l,k}\right)+\mu_{d,l,k}^{s,b_{S}}\left(s_{d,l,k}\right)-\mu_{d,l,k}^{s,b_{S}+}\left(s_{d,l,k}\right)\right)\right), \end{array}$$

where $\gamma_{d,l,k}^{b_{S}+,s}=\ln\frac{v_{d,l,k}^{b_{S}+,s}\left(s_{d,l,k}=1\right)}{v_{d,l,k}^{b_{S}+,s}\left(s_{d,l,k}=0\right)}$ is the log-likelihood ratio of $v_{d,l,k}^{b_{S}+,s}\left(s_{d,l,k}\right)$.

Based on the definitions in Equations (80), (101), and (102), the following log-likelihood ratio relationship can be derived:

(103) $$\begin{array}{c} \gamma_{d,l,k}^{s,b_{H}}=\left\{\begin{array}{c} \gamma_{d,l,k}^{b_{S},s}+\gamma_{d,l,k}^{b_{S}+,s},\;d=1,\cdots ,D-1,\\ \gamma_{d,l,k}^{b_{S},s},\;\;\;\;d=D. \end{array}\right. \end{array}$$

Then, using the definitions from (90), (99), and (102), the corresponding log-likelihood ratio relationship is expressed as

(104) $$\begin{array}{c} \gamma_{d,l,k}^{s,b_{S}}=\left\{\begin{array}{c} \gamma_{d,l,k}^{b_{H},s}+\gamma_{d,l,k}^{b_{S}+,s},\;d=1,\cdots ,D-1,\\ \gamma_{d,l,k}^{b_{H},s},\;\;\;\;d=D. \end{array}\right. \end{array}$$

Based on Equations (90), (100), and (101), the following log-likelihood ratio relationship can be further given:

(105) $$\begin{array}{c} \gamma_{d,l,k}^{s,b_{S}+}=\gamma_{d,l,k}^{b_{H},s}+\gamma_{d,l,k}^{b_{S},s},\;\;\;\;d=1,\cdots ,D-1. \end{array}$$

Following this, the marginal consistency constraint involving the variable belief $q_{S,d,l,k}\left(s_{d,l,k}\right)$ and the factor belief $b_{S,d,l,k}\left(s_{d,l,k},s_{d-1,l,k}\right)$ or $b_{S,1,l,k}\left(s_{1,l,k}\right)$ in (35) can be restated as

(106) $$\begin{array}{cc} \; & \gamma_{d,l,k}^{b_{S},s}\\ \; & =\left\{\begin{array}{c} \ln\frac{\rho_{l,k}^{1,0}}{\rho_{l,k}^{0,1}},\;\;\;\;\;\;\;\;\;d=1,\\ \ln\frac{\rho_{l,k}^{1,0}+\left(1-\rho_{l,k}^{0,1}\right)\exp\left(\gamma_{d-1,l,k}^{s,b_{S}+}\right)}{1-\rho_{l,k}^{1,0}+\rho_{l,k}^{0,1}\exp\left(\gamma_{d-1,l,k}^{s,b_{S}+}\right)},\;\;d=2,\cdots ,D. \end{array}\right. \end{array}$$

Moreover, when d=1,⋯,D−1, the marginal consistency constraint involving the beliefs $q_{S,d,l,k}\left(s_{d,l,k}\right)$ and $b_{S,d+1,l,k}\left(s_{d+1,l,k},s_{d,l,k}\right)$ in (35) can be rewritten as

(107) $$\begin{array}{c} \gamma_{d,l,k}^{b_{S}+,s}=\ln\frac{\rho_{l,k}^{0,1}+\left(1-\rho_{l,k}^{0,1}\right)\exp\left(\gamma_{d+1,l,k}^{s,b_{S}}\right)}{1-\rho_{l,k}^{1,0}+\rho_{l,k}^{1,0}\exp\left(\gamma_{d+1,l,k}^{s,b_{S}}\right)}. \end{array}$$

Subsequently, by taking the first-order partial derivative of the Lagrangian function (51) with respect to the transition probability $\rho_{l,k}^{0,1}$ and setting it to zero, $\rho_{l,k}^{0,1}$ can be expressed as the solution to the following quadratic equation:

(108) $$\begin{array}{c} A_{l,k}^{0,1}{\left(\rho_{l,k}^{0,1}\right)}^{2}+B_{l,k}^{0,1}\rho_{l,k}^{0,1}+C_{l,k}^{0,1}=0, \end{array}$$

where $A_{l,k}^{0,1}$, $B_{l,k}^{0,1}$, and $C_{l,k}^{0,1}$ are, respectively, given by   

(109) $$\begin{array}{cc} \; & A_{l,k}^{0,1}=E_{b_{S,1,l,k}}\left[s_{1,l,k}\right]-\sum_{d=2}^{D}E_{b_{S,d,l,k}}\left[s_{d-1,l,k}\right], \end{array}$$

(110) $$\begin{array}{cc} \; & B_{l,k}^{0,1}=\left(\rho_{l,k}^{1,0}-1\right)E_{b_{S,1,l,k}}\left[s_{1,l,k}\right]-\rho_{l,k}^{1,0}+\sum_{d=2}^{D}\{\left(1-\rho_{l,k}^{1,0}\right)\\ \; & \;\cdot E_{b_{S,d,l,k}}\left[s_{d-1,l,k}\right]-E_{b_{S,d,l,k}}\left[s_{d,l,k}s_{d-1,l,k}\right]\}, \end{array}$$

(111) $$\begin{array}{cc} \; & C_{l,k}^{0,1}=\rho_{l,k}^{1,0}\left(1-E_{b_{S,1,l,k}}\left[s_{1,l,k}\right]\right)\\ \; & +\rho_{l,k}^{1,0}\sum_{d=2}^{D}\left\{E_{b_{S,d,l,k}}\left[s_{d-1,l,k}\right]-E_{b_{S,d,l,k}}\left[s_{d,l,k}s_{d-1,l,k}\right]\right\}. \end{array}$$

Specifically, the solution for $\rho_{l,k}^{0,1}$ in (108) is expressed as

(112) $$\begin{array}{c} \rho_{l,k}^{0,1}=\frac{-B_{l,k}^{0,1}-\sqrt{{\left(B_{l,k}^{0,1}\right)}^{2}-4A_{l,k}^{0,1}C_{l,k}^{0,1}}}{2A_{l,k}^{0,1}}. \end{array}$$

Similarly, by taking the first-order partial derivative of the Lagrangian function (51) with respect to the transition probability $\rho_{l,k}^{1,0}$ and setting it to zero, $\rho_{l,k}^{1,0}$ can be expressed as the solution to the following quadratic equation:

(113) $$\begin{array}{c} A_{l,k}^{1,0}{\left(\rho_{l,k}^{1,0}\right)}^{2}+B_{l,k}^{1,0}\rho_{l,k}^{1,0}+C_{l,k}^{1,0}=0, \end{array}$$

where $A_{l,k}^{1,0}$, $B_{l,k}^{1,0}$, and $C_{l,k}^{1,0}$ are, respectively, given by

(114) $$\begin{array}{cc} A_{l,k}^{1,0} & =2-D-E_{b_{S,1,l,k}}\left[s_{1,l,k}\right]+\sum_{d=2}^{D}E_{b_{S,d,l,k}}\left[s_{d-1,l,k}\right], \end{array}$$

(115) $$\begin{array}{cc} B_{l,k}^{1,0} & =\left(1-\rho_{l,k}^{0,1}\right)E_{b_{S,1,l,k}}\left[s_{1,l,k}\right]-1-\rho_{l,k}^{0,1}\left(D-1\right)\\ \; & +\sum_{d=2}^{D}\{E_{b_{S,d,l,k}}\left[s_{d,l,k}\right]-E_{b_{S,d,l,k}}\left[s_{d,l,k}s_{d-1,l,k}\right]\\ \; & +\rho_{l,k}^{0,1}E_{b_{S,d,l,k}}\left[s_{d-1,l,k}\right]\}, \end{array}$$

(116) $$\begin{array}{cc} C_{l,k}^{1,0} & =\rho_{l,k}^{0,1}E_{b_{S,1,l,k}}\left[s_{1,l,k}\right]+\rho_{l,k}^{0,1}\\ \; & \cdot \sum_{d=2}^{D}\left\{E_{b_{S,d,l,k}}\left[s_{d,l,k}\right]-E_{b_{S,d,l,k}}\left[s_{d,l,k}s_{d-1,l,k}\right]\right\}, \end{array}$$

Specifically, the solution for $\rho_{l,k}^{1,0}$ in (113) can be calculated as

(117) $$\begin{array}{c} \rho_{l,k}^{1,0}=\frac{-B_{l,k}^{1,0}-\sqrt{{\left(B_{l,k}^{1,0}\right)}^{2}-4A_{l,k}^{1,0}C_{l,k}^{1,0}}}{2A_{l,k}^{1,0}}. \end{array}$$

Finally, considering the expression for the transition probability $\rho_{0,1}$ in (112) and the expression for the transition probability $\rho_{1,0}$ in (117), both are related to the mean of the factor belief $b_{S,d,l,k}\left(s_{d,l,k},s_{d-1,l,k}\right)$. When d=2,⋯,D, based on the factor belief $b_{S,d,l,k}\left(s_{d,l,k},s_{d-1,l,k}\right)$ in (65), we can derive the following series of mean expressions:   

(118) $$\begin{array}{cc} \; & E_{b_{S,d,l,k}}\left[s_{d,l,k}\right]=\frac{\psi_{d,l,k}^{1,0}+\psi_{d,l,k}^{1,1}}{\psi_{d,l,k}^{0,0}+\psi_{d,l,k}^{0,1}+\psi_{d,l,k}^{1,0}+\psi_{d,l,k}^{1,1}}, \end{array}$$

(119) $$\begin{array}{cc} \; & E_{b_{S,d,l,k}}\left[s_{d-1,l,k}\right]=\frac{\psi_{d,l,k}^{0,1}+\psi_{d,l,k}^{1,1}}{\psi_{d,l,k}^{0,0}+\psi_{d,l,k}^{0,1}+\psi_{d,l,k}^{1,0}+\psi_{d,l,k}^{1,1}}, \end{array}$$

(120) $$\begin{array}{cc} \; & E_{b_{S,d,l,k}}\left[s_{d,l,k}s_{d-1,l,k}\right]=\frac{\psi_{d,l,k}^{1,1}}{\psi_{d,l,k}^{0,0}+\psi_{d,l,k}^{0,1}+\psi_{d,l,k}^{1,0}+\psi_{d,l,k}^{1,1}}, \end{array}$$

where $\psi_{d,l,k}^{0,0}\overset{\Delta }{\;=}\left(1-\rho_{l,k}^{1,0}\right)\left(1-\rho_{d,l,k}^{s,b_{S}}\right)\left(1-\rho_{d-1,l,k}^{s,b_{S}+}\right)$, $\psi_{d,l,k}^{0,1}\overset{\Delta }{\;=}\left(\rho_{l,k}^{0,1}\right)\left(1-\rho_{d,l,k}^{s,b_{S}}\right)\left(\rho_{d-1,l,k}^{s,b_{S}+}\right)$, $\psi_{d,l,k}^{1,0}\overset{\Delta }{\;=}\left(\rho_{l,k}^{1,0}\right)\left(\rho_{d,l,k}^{s,b_{S}}\right)\left(1-\rho_{d-1,l,k}^{s,b_{S}+}\right)$, $\psi_{d,l,k}^{1,0}\overset{\Delta }{\;=}\left(1-\rho_{l,k}^{0,1}\right)\left(\rho_{d,l,k}^{s,b_{S}}\right)\left(\rho_{d-1,l,k}^{s,b_{S}+}\right)$, $\rho_{d,l,k}^{s,b_{S}}=1/\left(1+\exp\left(-\gamma_{d,l,k}^{s,b_{S}}\right)\right)$, and $\rho_{d,l,k}^{s,b_{S}+}=1/\left(1+\exp\left(-\gamma_{d,l,k}^{s,b_{S}+}\right)\right)$. When d=1, based on the factor belief $b_{S,1,l,k}\left(s_{1,l,k}\right)$ in (66), the mean expression can be obtained as

(121) $$\begin{array}{c} E_{b_{S,1,l,k}}\left[s_{1,l,k}\right]=\frac{\rho_{l,k}^{1,0}\rho_{1,l,k}^{s,b_{S}}}{\rho_{l,k}^{1,0}\rho_{1,l,k}^{s,b_{S}}+\rho_{l,k}^{0,1}\left(1-\rho_{1,l,k}^{s,b_{S}}\right)}, \end{array}$$

where $\rho_{1,l,k}^{s,b_{S}}=1/\left(1+\exp\left(-\gamma_{1,l,k}^{s,b_{S}}\right)\right)$ denotes the probability that the variable $s_{1,l,k}$ equals 1, with the corresponding log-likelihood ratio given by $\gamma_{1,l,k}^{s,b_{S}}$.

### 5.7. Channel Value Module

This subsection will provide a detailed description of the message passing process in the channel value module. Based on the mean expression for the variable belief $q_{G,j}\left(g_{j}\right)$ in (67), we have $\tau_{j}^{g,b_{H}}=-\tau_{j}^{g,b_{G}}$. For the factor belief $b_{\tilde{G},j}\left(g_{j}\right)$ in (68), by expanding and simplifying the term $f_{G,j}\left(g_{j},\omega_{j}\right)$, the following can be obtained:

(122) $$\begin{array}{cc} b_{\tilde{G},j}\left(g_{j}\right) & \propto \mathrm{CN}\left(g_{j};\xi_{j}^{g,b_{G}},1/\eta_{j}^{g,b_{\tilde{G}}}\right)\mathrm{CN}\left(g_{j};0,1/\eta_{j}^{g,b_{\Omega }}\right)\\ \; & \propto \mathrm{CN}\left(g_{j};\frac{\xi_{j}^{g,b_{\tilde{G}}}\eta_{j}^{g,b_{\tilde{G}}}}{\eta_{j}^{g,b_{\tilde{G}}}+\eta_{j}^{g,b_{\Omega }}},\frac{1}{\eta_{j}^{g,b_{\tilde{G}}}+\eta_{j}^{g,b_{\Omega }}}\right), \end{array}$$

where $\eta_{j}^{g,b_{\Omega }}=\sum_{j^{\prime }\in N_{j}}E_{b_{\Omega ,j^{\prime }}}\left[\omega_{j^{\prime }}\right]$. By substituting the variable belief $q_{G,j}\left(g_{j}\right)$ from (67) and the factor belief $b_{\tilde{G},j}\left(g_{j}\right)$ from (122) into the variance consistency constraint ${\mathrm{Var}}_{q_{G,j}}\left[g_{j}\right]={\mathrm{Var}}_{b_{\tilde{G},j}}\left[g_{j}\right]$ in (49), we have

(123) $$\begin{array}{c} \eta_{j}^{g,b_{H}}=\eta_{j}^{g,b_{\Omega }}=\sum_{j^{\prime }\in N_{j}}E_{b_{\Omega ,j^{\prime }}}\left[\omega_{j^{\prime }}\right]. \end{array}$$

By then substituting $\tau_{j}^{g,b_{H}}=-\tau_{j}^{g,b_{\tilde{G}}}$ and $\eta_{j}^{g,b_{H}}=\eta_{j}^{g,b_{\Omega }}$ into (122), and combining the mean consistency constraint $E_{b_{H,j}}\left[g_{j}\right]=E_{b_{\tilde{G},j}}\left[g_{j}\right]$ from (48) with the definition of the auxiliary mean $\xi_{j}^{g,b_{H}}$ in (61), after simplification, it follows that the mean of the factor belief $b_{\tilde{G},j}\left(g_{j}\right)$ is given by $E_{b_{H,j}}\left[g_{j}\right]=-\tau_{j}^{g,b_{H}}/\eta_{j}^{g,b_{H}}$. Consequently, the auxiliary mean $\xi_{j}^{g,b_{H}}$ can be expressed as

(124) $$\begin{array}{c} \xi_{j}^{g,b_{H}}=E_{b_{\tilde{G},j}}\left[g_{j}\right]+\frac{\tau_{j}^{g,b_{H}}}{\eta_{j}^{g,b_{H}}}=0. \end{array}$$

Next, by expanding and simplifying the term $f_{G,j}\left(g_{j},\omega_{j}\right)$ in the factor belief $b_{\Omega ,j}\left(\omega_{j}\right)$ from (69), the following expression can be obtained:

(125) $$\begin{array}{cc} \; & b_{\Omega ,j}\left(\omega_{j}\right)\propto f_{\Omega ,j}\left(\omega_{j}\right)\\ \; & \;\cdot \exp\left(\varpi \left(\omega_{j}\right)-\omega_{j}\left(\sum_{j^{\prime }\in N_{j}}E_{b_{\tilde{G},j^{\prime }}}\left[{\left|g_{j^{\prime }}\right|}^{2}\right]\right)\right), \end{array}$$

where $\varpi_{j}\left(\omega_{j}\right)$ is given by

(126) $$\begin{array}{c} \varpi \left(\omega_{j}\right)=\sum_{j^{\prime }\in N_{j}}\int \ln\left(\omega_{j}+\sum_{j^{\prime \prime }\in N_{j^{\prime }\setminus j}}\omega_{j^{\prime \prime }}\right)d\omega_{j^{\prime }\setminus j}. \end{array}$$

As stated in (24), we have $f_{\Omega ,j}\left(\omega_{j}\right)=\mathrm{Ga}\left(\omega_{j};a,b\right)$. To simplify the calculation, it is desirable for the $\exp\left(\cdot \right)$ function in (125) to be proportional to a Gamma distribution so that $b_{\Omega ,j}\left(\omega_{j}\right)$ also follows a Gamma distribution, which would facilitate the computation of the mean $E_{b_{\Omega ,j}}\left[\omega_{j}\right]$. However, due to the presence of variable coupling in the $\ln\left(\cdot \right)$ function, obtaining a closed-form solution for $\varpi \left(\omega_{j}\right)$ in (126) is very challenging. Notably, the $\exp\left(\cdot \right)$ function in (125) contains both linear and nonlinear parts in $\omega_{j}$, which are coupled with other variables. We refer to this as the fully coupled model. Next, consider an alternative scenario where all $\omega_{j^{\prime }}$ in the prior (23) are replaced by ωj, leading to $f_{G,j}\left(g_{j},\omega_{j}\right)=\mathrm{CN}\left(g_{j};0,{\left(\sum_{j^{\prime }\in N_{j}}\omega_{j}\right)}^{-1}\right)=\mathrm{CN}\left(g_{j};0,{\left(\left|N_{j}\right|\omega_{j}\right)}^{-1}\right)$. In this case, we can derive

(127) $$\begin{array}{cc} \; & b_{\Omega ,j}\left(\omega_{j}\right)\propto f_{\Omega ,j}\left(\omega_{j}\right)\\ \; & \;\;\cdot \exp\left(\ln\left(\left|N_{j}\right|\omega_{j}\right)-\omega_{j}\left|N_{j}\right|E_{b_{G,j}}\left[{\left|g_{j}\right|}^{2}\right]\right), \end{array}$$

which we refer to as the decoupled model. To strike a balance between computational convenience and variable correlation, we can retain the linear part of the $\exp\left(\cdot \right)$ function in $b_{\Omega ,j}\left(\omega_{j}\right)$ under the fully coupled mode, while replacing the nonlinear part $\varpi_{j}\left(\omega_{j}\right)$ with the nonlinear part $\ln\left(\left|N_{j}\right|\omega_{j}\right)$ from the uncoupled mode, thus obtaining

(128) $$\begin{array}{cc} \; & b_{\Omega ,j}\left(\omega_{j}\right)\propto f_{\Omega ,j}\left(\omega_{j}\right)\\ \; & \cdot \exp\left(\ln\left(\left|N_{j}\right|\omega_{j}\right)-\omega_{j}\left(\sum_{j^{\prime }\in N_{j}}E_{b_{\tilde{G},j^{\prime }}}\left[{\left|g_{j^{\prime }}\right|}^{2}\right]\right)\right)\\ \; & \propto \mathrm{Ga}\left(\omega_{j};a+1,b+\sum_{j^{\prime }\in N_{j}}E_{b_{\tilde{G},j^{\prime }}}\left[{\left|g_{j^{\prime }}\right|}^{2}\right]\right). \end{array}$$

where the factor belief $b_{\Omega ,j}\left(\omega_{j}\right)$ after the replacement is referred to as the semi-coupled model. The mean of this semi-coupled factor belief can be computed as follows:

(129) $$\begin{array}{cc} E_{b_{\Omega ,j}}\left[\omega_{j}\right] & =\frac{a+1}{b+\sum_{j^{\prime }\in N_{j}}E_{b_{\tilde{G},j^{\prime }}}\left[{\left|g_{j^{\prime }}\right|}^{2}\right]}\\ \; & =\frac{a+1}{b+\sum_{j^{\prime }\in N_{j}}E_{b_{H,j^{\prime }}}\left[{\left|g_{j^{\prime }}\right|}^{2}\right]}, \end{array}$$

where the second equality arises due to the consistency constraint between the mean and variance, which implies that $E_{b_{H,j}}\left[{\left|g_{j}\right|}^{2}\right]=E_{b_{\tilde{G},j}}\left[{\left|g_{j}\right|}^{2}\right]$, and $E_{b_{H,j}}\left[{\left|g_{j}\right|}^{2}\right]$ is given by (93).

### 5.8. Active User Detection Strategy

This section will provide a detailed description of the active user detection strategy. After obtaining the estimated values of the effective angle–delay domain channel ${\hat{h}}_{j}^{\mathrm{ad}}=E_{b_{H,j}}\left[h_{j}\right],\forall j$ (i.e., ${\hat{h}}_{d,l,k}^{\mathrm{ad}}=E_{b_{H,d,l,k}}\left[h_{d,l,k}\right],\forall d,l,k$), the equivalent space–frequency domain channel estimate for user *k* can be derived based on the channel transformation relationship in (9) as

(130) $$\begin{array}{c} {\hat{H}}_{k}^{\mathrm{sf}}=A{\hat{H}}_{k}^{\mathrm{ad}}B^{T}, \end{array}$$

where ${\hat{h}}_{d,l,k}^{\mathrm{ad}}$ represents the (d,l)-th element of the matrix ${\hat{H}}_{k}^{\mathrm{ad}}$. The algorithm determines the active status of user *k* using a detector based on the user’s activity log-likelihood ratio, as described in [32,33,34], and this is expressed as

(131) $$\begin{array}{c} {\hat{\alpha }}_{k}=\left\{\begin{array}{c} 1,\;\;\;\gamma_{k}^{b_{A}}>0,\\ 0,\;\;\;\gamma_{k}^{b_{A}}\leq 0, \end{array}\right. \end{array}$$

where the update for $\gamma_{k}^{b_{A}}$ is given in (98). Then, based on the activity detection metric ${\hat{\alpha }}_{k}$, the equivalent space–frequency domain channel estimation for user *k* is adjusted as

(132) $$\begin{array}{c} {\hat{H}}_{k}^{\mathrm{sf}}=\left\{\begin{array}{c} {\hat{H}}_{k}^{\mathrm{sf}},\;\;\;\alpha_{k}=1,\\ \;0,\;\;\;\;\;\;\;\alpha_{k}=0. \end{array}\right. \end{array}$$

Finally, the equivalent space–frequency domain channel estimation matrix for all users can be obtained as ${\hat{H}}^{\mathrm{sf}}=\left[{\hat{H}}_{1}^{\mathrm{sf}},{\hat{H}}_{2}^{\mathrm{sf}},\cdots ,{\hat{H}}_{K}^{\mathrm{sf}}\right]$, along with the activity detection vector $\hat{\alpha }={\left[{\hat{\alpha }}_{1},{\hat{\alpha }}_{2},\cdots ,{\hat{\alpha }}_{K}\right]}^{T}$.

## 6. Algorithm Description

### 6.1. Procedure Details

The detailed procedure of the proposed CHMP algorithm is summarized in Algorithm 1. In this algorithm, the equivalent channel module, serving as a connecting component, is divided into its corresponding modules. The Markov chain of the channel support module corresponds to Steps 17 to 24 of the algorithm, while the coupled Gaussian distribution in the channel value module corresponds to Steps 32 and 33. The algorithm will iterate according to the rules until convergence is achieved.

The CHMP algorithm adopts an EM-like iterative update strategy and utilizes the damping technique to enhance the convergence stability [35,36,37]. Every $\Delta_{T}$ iterations, the algorithm executes the update steps corresponding to the aforementioned correlated probability model. In the remaining iterations, the channel support module executes the update Step 26, which corresponds to the i.i.d. Bernoulli prior

(133) $$\begin{array}{c} \gamma_{d,l,k}^{s,b_{H}}=\gamma_{d,l,k}^{b_{H},s}+\gamma_{d,l,k}^{s,b_{H}\left(\mathrm{old}\right)}, \end{array}$$

The channel value module executes Step 35, which corresponds to the i.i.d. zero-mean complex Gaussian prior

(134) $$\begin{array}{c} \eta_{j}^{g,b_{H}}=1/E_{b_{H,j}}\left[{\left|g_{j}\right|}^{2}\right]. \end{array}$$

### 6.2. Computational Complexity

Considering that Step 6 of the CHMP algorithm involves a matrix–vector product of the form $\Phi_{\circ i}u$, where $\Phi_{\circ i}\in C^{R\times J}$ (with R=MPG and J=DLK), and *i* can take the value 1 or 2, we define $\Phi_{\circ 1}=\Phi$ and $\Phi_{\circ 2}$ as the matrix obtained by squaring the modulus of each element in Φ. Storing the matrix $\Phi_{\circ i}$ is computationally expensive, and calculating this product $\Phi_{\circ i}u$ requires MPGDLK multiplications and MPG(DLK−1) additions. According to (13), we have $\Phi_{\circ i}={\tilde{B}}_{\circ i}^{T}⊗A_{\circ i}$, where $A_{\circ i}\in C^{M\times D}$ and ${\tilde{B}}_{\circ i}\in C^{LK\times PG}$. Using this relationship, the matrix–vector product can be rewritten as

(135) $$\begin{array}{c} \Phi_{\circ i}u=\mathrm{vec}\left(A_{\circ i}U{\tilde{B}}_{\circ i}\right), \end{array}$$

where $U\in C^{D\times LK}$, and $u=\mathrm{vec}\left(U\right)$. By left-multiplying U with $A_{\circ i}$ and then right-multiplying with ${\tilde{B}}_{\circ i}$, the computational complexity is reduced to $M\left(PG+D\right)LK$ multiplications and $\left(M\left(PG+D-1\right)LK-MPG\right)$ additions. Alternatively, by  right-multiplying U with ${\tilde{B}}_{\circ i}$ and then left-multiplying with $A_{\circ i}$, the computational complexity is reduced to $D\left(LK+M\right)PG$ multiplications and $\left(D\left(LK+M-1\right)PG-MPG\right)$ additions. Therefore, depending on the specific system configuration, the optimal implementation method can be selected to minimize the computational cost.

Additionally, Step 8 involves a matrix–vector product of the form $\Phi_{\circ i}^{H}v$, which can similarly be computed using the following relation:

(136) $$\begin{array}{c} \Phi_{\circ i}^{H}v=\mathrm{vec}\left(A_{\circ i}^{H}V{\tilde{B}}_{\circ i}^{H}\right), \end{array}$$

where $V\in C^{M\times PG}$, and $v=\mathrm{vec}\left(V\right)$. If we first right-multiply V by ${\tilde{B}}_{\circ i}^{H}$ and then left-multiply by $A_{\circ i}^{H}$, the computational complexity is reduced to $M\left(PG+D\right)LK$ multiplications and $\left(M\left(PG+D-1\right)LK-DLK\right)$ additions. Alternatively, if we first left-multiply V by $A_{\circ i}^{H}$ and then right-multiply by ${\tilde{B}}_{\circ i}^{H}$, the computational complexity is reduced to $D\left(LK+M\right)PG$ multiplications and $\left(D\left(LK+M-1\right)PG-DLK\right)$ additions. Thus, depending on the specific system configuration, the implementation method with the minimal computational cost can be chosen.

Next, the computational complexity is assessed using real-valued floating-point operations (FLOPs), where the FLOP cost is determined by the type of operation. Specifically, addition, subtraction, multiplication, division, and the square root of two real numbers each require one FLOP; the addition and subtraction of two complex numbers each require two FLOPs; the multiplication and division of a complex number by a real number each require two FLOPs; the square of the modulus of a complex number requires three FLOPs; and multiplying two complex numbers requires six FLOPs (excluding complex conjugation). Additionally, the $\exp\left(\cdot \right)$ and $\ln\left(\cdot \right)$ functions for real numbers can be implemented using lookup tables, which does not consume computational resources [38,39]. Consequently, the total computational complexity of the CHMP algorithm is $O\left(T\cdot \min\left\{M\left(PG+D\right)LK,D\left(LK+M\right)PG\right\}\right)$.
**Algorithm 1** CHMP  1:**Input**: A, B, $\tilde{B}$, y, σ, $a=b=10^{-20}$, $\Delta_{T}=2$, ϑ=0.7.  2:**Initialize**: $\tau_{r}^{z,b_{Y}}=\eta_{j}^{h,b_{H}}=\gamma_{j}^{\alpha ,b_{H}}=\gamma_{j}^{s,b_{H}}=\gamma_{j}^{b_{S},s}=\gamma_{j}^{b_{S}+,s}=0$, $\rho_{k}=0.5$, $\rho_{l,k}^{0,1}=\rho_{l,k}^{1,0}=0.5$, $E_{b_{H,j}}\left[h_{j}\right]=0$, ${\mathrm{Var}}_{b_{H,j}}\left[h_{j}\right]=\frac{\rho_{l,k}^{0,1}\rho_{k}\left({∥y∥}_{F}^{2}-R\sigma \right)}{{∥A∥}_{F}^{2}{∥\tilde{B}∥}_{F}^{2}}$, ∀l,k,r,j.  3:**for** t=1,⋯,T **do**  4:     // Signal Transmission  5:    Update the auxiliary precision $\eta_{j}^{h,b_{Z}}$ according to (72), ∀j.  6:    Update the auxiliary precision $\eta_{r}^{z,b_{Y}}$ and the auxiliary mean $\xi_{r}^{z,b_{Y}}$ according to (73) and (74), ∀r.  7:    Update the auxiliary multiplier $\tau_{r}^{z,b_{Y}}$ according to (75), ∀r.  8:    Update the auxiliary precision $\eta_{j}^{h,b_{H}}$ and the auxiliary mean $\xi_{j}^{h,b_{H}}$ according to (77) and (78), ∀j.  9:     // Activity Indicator10:    Update the log-likelihood ratio $\gamma_{j}^{b_{H},\alpha }$ according to (89), ∀j.11:    Update the log-likelihood ratio $\gamma_{k}^{\alpha ,b_{A}}$ according to (95), ∀k.12:    Update the user activity log-likelihood ratio $\gamma_{k}^{b_{A}}$ according to (98), ∀k.13:    Update the log-likelihood ratio $\gamma_{d,l,k}^{\alpha ,b_{H}}$ using (96), ∀d,l,k.14:     // Channel Support15:    Update the log-likelihood ratio $\gamma_{d,l,k}^{b_{H},s}$ using (91), ∀d,l,k.16:    **if** *t* is an integer multiple of $\Delta_{T}$ **then**17:        Update the log-likelihood ratios $\gamma_{d,l,k}^{s,b_{S}}$ and $\gamma_{d,l,k}^{s,b_{S}+}$ according to (104) and (105), ∀d,l,k.18:        Update the mean $E_{b_{S,d,l,k}}\left[s_{d,l,k}\right]$ according to (118) and (121), ∀d,l,k.19:        Update the mean $E_{b_{S,d,l,k}}\left[s_{d-1,l,k}\right]$ according to (119), d=2,⋯,D, ∀l,k.20:        Update the mean $E_{b_{S,d,l,k}}\left[s_{d,l,k}s_{d-1,l,k}\right]$ according to (120), d=2,⋯,D, ∀l,k.21:        Update the probability $\rho_{l,k}^{0,1}$ using (109)–(112), ∀l,k.22:        Update the probability $\rho_{l,k}^{1,0}$ using (114)–(117), ∀l,k.23:        Update the log-likelihood ratios $\gamma_{d,l,k}^{b_{S},s}$ and $\gamma_{d,l,k}^{b_{S}+,s}$ according to (106) and (107), ∀d,l,k.24:        Update the log-likelihood ratio $\gamma_{d,l,k}^{s,b_{H}}$ using (103), ∀d,l,k.25:    **else**26:        Update the log-likelihood ratio $\gamma_{d,l,k}^{s,b_{H}}$ using (133), ∀d,l,k.27:    **end if**28:     // Channel Value29:    Update the non-zero probability ${\tilde{\rho }}_{j}^{b_{H}}$, the Gaussian mean ${\tilde{\xi }}_{j}^{b_{H}}$, and the Gaussian variance ${\tilde{\lambda }}_{j}^{b_{H}}$ according to (83)–(85), ∀j.30:    Update the mean square $E_{b_{H,j}}\left[{\left|g_{j}\right|}^{2}\right]$ according to (93), ∀j.31:    **if** *t* is an integer multiple of $\Delta_{T}$ **then**32:        Update the mean $E_{b_{\Omega ,j}}\left[\omega_{j}\right]$ according to (129), ∀j.33:        Update the precision $\eta_{j}^{g,b_{H}}$ according to (123), ∀j.34:    **else**35:        Update the precision $\eta_{j}^{g,b_{H}}$ according to (134), ∀j.36:    **end if**37:    Update the mean $E_{b_{H,j}}\left[h_{j}\right]$ and the variance ${\mathrm{Var}}_{b_{H,j}}\left[h_{j}\right]$ according to (86) and (87), ∀j.38:**end for**39:Detect the user activity ${\hat{\alpha }}_{k}$ and obtain the equivalent space–frequency domain channel ${\hat{H}}_{k}^{\mathrm{sf}}$ according to (130)–(132), ∀k.40:**Output**: The equivalent space–frequency domain channel estimation ${\hat{H}}^{\mathrm{sf}}$ and the activity detection $\hat{\alpha }$ for all users.

## 7. Simulation Results

This section presents the simulation results and analysis of the CHMP algorithm. The channel parameters in the simulation were generated using the QuaDRiGa platform [40], with the simulation scenario set to ‘3GPP_38.901_UMa_NLOS’ [41]. The simulation parameters are shown in Table 3. This work employs the Normalized Mean Square Error (NMSE) and Activity Error Rate (AER) as performance metrics to evaluate the accuracy of channel estimation and active user detection, respectively. The NMSE and AER are defined as follows:

(137) $$\begin{array}{cc} \mathrm{NMSE} & =\frac{1}{M_{c}}\sum_{m_{c}=1}^{M_{c}}\frac{∥{\hat{H}}_{\left(m_{c}\right)}^{\mathrm{sf}}-H_{\left(m_{c}\right)}^{\mathrm{sf}}{∥}_{F}^{2}}{∥H_{\left(m_{c}\right)}^{\mathrm{sf}}{∥}_{F}^{2}}, \end{array}$$

(138) $$\begin{array}{cc} \mathrm{AER} & =\frac{1}{M_{c}}\sum_{m_{c}=1}^{M_{c}}\frac{{∥{\hat{\alpha }}_{\left(m_{c}\right)}-\alpha_{\left(m_{c}\right)}∥}_{1}}{K}, \end{array}$$

where ${∥\cdot ∥}_{F}$ and ${∥\cdot ∥}_{1}$ denote the Frobenius norm and $\ell_{1}$ norm, respectively, and Mc represents the number of Monte Carlo trials. To ensure a high degree of statistical reliability and to minimize the impact of random fluctuations, all subsequent simulation results are averaged over 1000 independent Monte Carlo trials, which is consistent with [14,19]. This large number of repetitions is standard practice for performance evaluation in this field and ensures that the presented curves are stable and robust estimates of the true performance.

The baseline algorithms compared in the simulation include (1) the CHMP algorithm with channel support correlation only, which does not consider channel value correlation, where channel values $g_{j}$ follow a complex Gaussian distribution with mean 0 and variance $\omega_{j}^{-1}$; (2) the CHMP algorithm with channel value correlation only, which does not consider channel support correlation, where channel support $s_{j}$ follows a Bernoulli distribution with $\mathrm{Pr}\left(s_{j}=1\right)=\rho_{j}$; (3) the CHMP algorithm without correlation, which does not consider either channel support or value correlation; (4) the GGAMP-SBL algorithm in [42], which combines SBL with the Gaussian GAMP framework to achieve robust performance; (5) the hybrid EM-VMP-EP variant algorithm in [43], which uses expectation–maximization (EM) to learn model parameters, variational message passing (VMP) to decouple the estimation of parameters, and an EP variant to perform sparse signal recovery; (6) the HMP-DCT algorithm in [13], which combats frequency-selective fading by first employing the discrete cosine transform (DCT) to create a sparse representation of the channel and then performs joint UAD and CE using an efficient hybrid message passing (HMP) algorithm, where the prior of the DCT coefficients is modeled with a Cauchy distribution and its unknown hyperparameters are learned via a gradient descent method.

### 7.1. Impact of SNR

**Figure 3:**
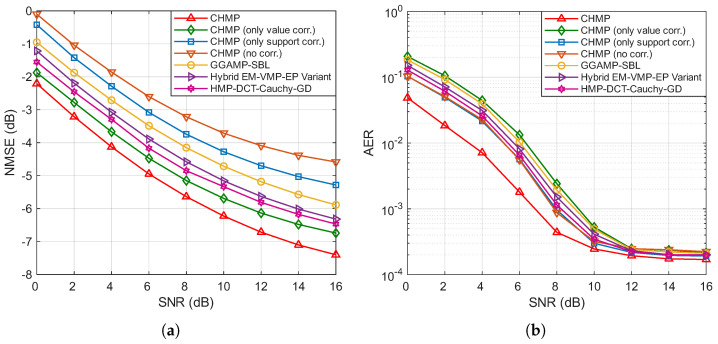
(**a**) NMSE performance of different algorithms under various SNRs. (**b**) AER performance of different algorithms under various SNRs.

Figure 3a,b illustrate the variation in the NMSE and AER with respect to the SNR for different algorithms, with the number of BS antennas *M* set to 64 and the number of active users $K_{a}$ set to 60. It can be observed that the NMSE and AER of all algorithms decrease as the SNR increases. Specifically, the rate of NMSE reduction for all algorithms gradually slows as the SNR increases, with all curves exhibiting smooth and consistent downward trends. For the AER, starting from 0 dB, the values initially decrease rapidly with increasing SNRs, but, beyond 10 or 12 dB, further increases in the SNR result in only marginal reductions in the AER. From the performance comparison of the algorithms, it is evident that the CHMP algorithm, which simultaneously considers both channel support and value correlations, achieves the best performance in both channel estimation and active user detection.

In the NMSE curves shown in Figure 3a, compared to the CHMP algorithm without correlation, incorporating the correlation of channel values using a coupled Gaussian distribution and modeling channel support correlation with a Markov chain both enhance algorithm performance. The performance improvement from channel support correlation is relatively modest, while the channel value correlation yields a more substantial improvement. The joint utilization of both correlations results in the greatest performance gain. Among the baseline algorithms, HMP-DCT-Cauchy-GD demonstrates strong performance, consistently outperforming the general-purpose GGAMP-SBL and hybrid EM-VMP-EP variant across the entire SNR range. This highlights the effectiveness of using the discrete cosine transform to exploit channel sparsity. However, our proposed CHMP algorithm and the CHMP with only value correlation still maintain a clear performance gap regarding HMP-DCT-Cauchy-GD. This underscores the substantial benefit of our proposed dual-correlation channel model, which provides a more accurate channel prior than the models implicitly or explicitly used in these advanced algorithms.

In the AER curves shown in Figure 3b, the CHMP algorithm again demonstrates the best performance. Notably, in the low-to-medium SNR range (0 dB to 8 dB), the proposed CHMP algorithm demonstrates clear and substantial superiority in the AER. In this range, HMP-DCT-Cauchy-GD achieves competitive AER performance, which is better than that of the hybrid EM-VMP-EP variant but slightly worse than that of the CHMP algorithm with only support correlation. GGAMP-SBL and the hybrid EM-VMP-EP variant, in turn, outperform CHMP with only value correlation, but their performance is worse than that of CHMP with only support correlation and CHMP without correlation. As the SNR increases into the high regime (above 10 dB), the performance improvement for all algorithms becomes marginal. Here, the performance of GGAMP-SBL becomes similar to that of CHMP with only value correlation and CHMP without correlation, while the hybrid EM-VMP-EP variant performs comparably to CHMP with only support correlation. However, all these algorithms remain slightly inferior to the proposed CHMP algorithm, which maintains the lowest AER. Furthermore, CHMP with only value correlation shows less competitive AER performance, primarily because the coupled Gaussian distribution affects the judgment of activity indicators. Furthermore, it can be seen that the Markov chain mitigates the negative impact of the coupled Gaussian distribution on the AER. Their joint modeling effectively captures the internal structure of channel support and the inherent correlation of channel values, leading to the most significant performance improvement.

### 7.2. Impact of the Number of Active Users

**Figure 4:**
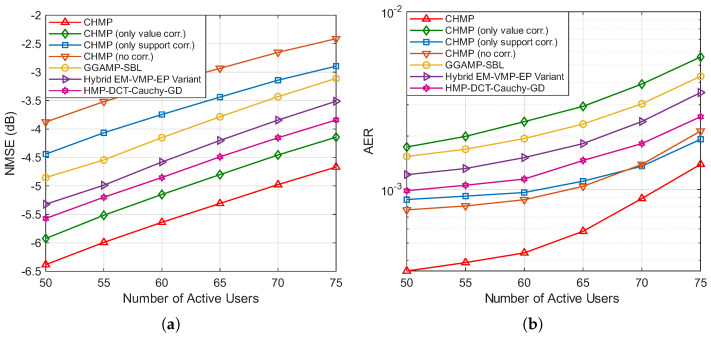
(**a**) NMSE performance of different algorithms under various numbers of active users. (**b**) AER performance of different algorithms under various numbers of active users.

Figure 4a,b illustrate the NMSE and AER performance of various algorithms as a function of the number of active users, with the number of BS antennas *M* set to 64 and the SNR set to 8 dB. It is observed that the performance of all algorithms degrades as the number of active users grows, increasing the difficulty of the sparse recovery problem.

Specifically, in the NMSE curves shown in Figure 4a, compared to the CHMP algorithm without considering correlations, utilizing channel value correlation yields a more significant improvement than channel support correlation, and the joint utilization of both results in the greatest performance enhancement. Notably, the proposed CHMP algorithm maintains a clear performance advantage over all baselines. Among these, HMP-DCT-Cauchy-GD delivers a better NMSE than both GGAMP-SBL and the hybrid EM-VMP-EP variant across the entire range of user loads. In the AER curves shown in Figure 4b, the superiority of the proposed CHMP algorithm is even more pronounced. The HMP-DCT-Cauchy-GD algorithm’s AER performance is competitive, surpassing the hybrid EM-VMP-EP variant and performing just below CHMP with only support correlation. The curves for the algorithm without correlations and the one considering only channel support correlation are quite similar. GGAMP-SBL and the hybrid EM-VMP-EP variant are superior to CHMP with only value correlation but are clearly outperformed by the other three CHMP algorithms. The coupled Gaussian distribution, which is used in CHMP with only value correlation, has a negative impact on the AER. The proposed CHMP algorithm, which jointly utilizes both support and value correlations, consistently maintains the lowest NMSE and AER across all tested user loads. This demonstrates not only the effectiveness but also the robustness of our method, as its performance advantage is sustained over all baselines, even under the increasingly difficult conditions of a crowded massive access scenario. This again suggests that our tailored dual-correlation model provides a more accurate representation of the channel’s physical structure than the general-purpose sparsity assumption exploited by the HMP-DCT-Cauchy-GD algorithm.

### 7.3. Impact of the Number of BS Antennas

**Figure 5:**
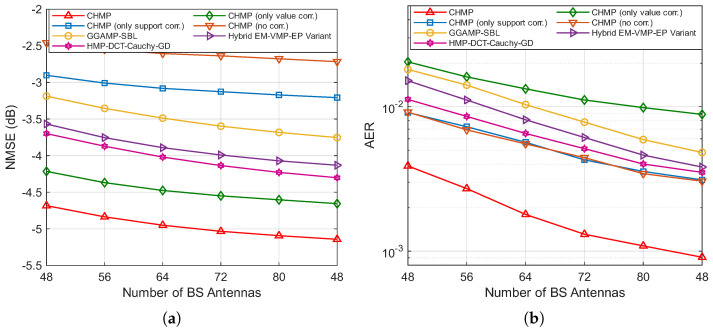
(**a**) NMSE performance of different algorithms under various numbers of BS antennas. (**b**) AER performance of different algorithms under various numbers of BS antennas.

Figure 5a,b illustrate the NMSE and AER performance as a function of the number of BS antennas, with the number of active users $K_{a}$ set to 60 and the SNR set to 6 dB. As expected, the performance of all algorithms improves as the number of antennas increases, owing to the enhanced spatial resolution and array gain.

In the NMSE curves, the performance hierarchy remains consistent, with the full CHMP algorithm and the CHMP algorithm with only value correction leading. The HMP-DCT-Cauchy-GD algorithm performs well, showing a better NMSE than the other baselines. However, the performance gap between our proposed CHMP algorithm and all other methods, including HMP-DCT-Cauchy-GD, remains significant and even appears to widen slightly as the number of antennas grows. This trend is particularly revealing. In the AER curves shown in Figure 5b, the proposed CHMP algorithm again shows a significant advantage over all other methods. The HMP-DCT-Cauchy-GD algorithm’s AER is competitive and better than that of the hybrid EM-VMP-EP variant, while being slightly outperformed by CHMP with only support correlation and CHMP without correlation. This again highlights that joint correlation modeling effectively captures the internal structure of channel support and the inherent relationships of channel values. The widening performance gap demonstrates that our angle–delay domain dual-correlation model is exceptionally effective at leveraging the additional spatial information afforded by larger massive MIMO arrays. While HMP-DCT-Cauchy-GD effectively handles frequency-domain structures, our approach’s explicit modeling of the spatial channel characteristics allows it to better capitalize on the increase in antenna elements, leading to more accurate detection and estimation.

## 8. Conclusions

This paper investigates the joint active user detection and channel estimation problem in the uplink grant-free random access scenario of massive MIMO-OFDM systems. First, we propose an effective probability model that accurately characterizes the sparse nature of the massive random access channel in the user–angle–delay domain by introducing three types of state variables: active indicators, channel supports, and channel values. The proposed probability model not only captures the sparsity of the channel in the user domain but also leverages Markov chains and coupled Gaussian distributions to deeply explore the correlation properties of the channel in the angle–delay domain. Then, based on this probability model, the joint active user detection and channel estimation problem is formulated as a BFE minimization problem under hybrid constraints. To address this problem effectively, we further propose the CHMP algorithm, which adaptively adjusts the model parameters under unknown user sparsity and channel prior information. Numerical simulations validate the advantages of the proposed method in active user detection and channel estimation. While this work provides a robust framework for joint detection and estimation, we acknowledge that our analysis is based on a quasi-static channel model. In practical grant-free scenarios, impairments such as channel aging due to user mobility and imperfect synchronization can affect performance. Extending the proposed CHMP algorithm to account for these dynamic, non-ideal conditions and evaluating its robustness is an important and valuable direction for future research.

## Figures and Tables

**Table 1:** Table of notations.

Symbol	Definition
System Parameters	
*M*	Number of antennas at the BS
*K*	Number of potential users
$K_{a}$	Number of active users
*N*	Number of effective subcarriers
*G*	Number of OFDM symbols for access
*P*	Number of pilot subcarriers
D,L	Number of grid points in angle and delay domains
Channel and Signal Representations	
$\alpha_{k}$	Activity indicator for user *k*
$h_{n,k}^{\mathrm{SF}}$	Space–frequency domain channel vector for user *k* on subcarrier *n*
$H_{k}^{\mathrm{SF}}$	Space–frequency domain channel matrix for user *k*
$H_{k}^{\mathrm{AD}}$	Angle–delay domain channel matrix for user *k*
$H_{k}^{\mathrm{sf}},H_{k}^{\mathrm{ad}}$	Equivalent channels (multiplied by activity indicator $\alpha_{k}$)
y,Y	Received vectorized signal and matrix signal
h	Vectorized equivalent angle–delay domain channel for all users
Φ	Sensing matrix
Probabilistic Model Variables	
$S_{k}$	Binary channel support matrix for user *k*
$G_{k}$	Channel value matrix for user *k*
$\rho_{k}$	Activity probability of user *k*
$\rho_{l,k}^{0,1},\rho_{l,k}^{1,0}$	Transition probabilities of the Markov chain for channel support
$\omega_{d,l,k}$	Latent precision of the coupled Gaussian distribution
$\Omega_{k}$	Latent precision matrix for user *k*
γ,τ,η,κ,μ	Lagrange multipliers for different constraints

**Table 2:** Factor beliefs and variable beliefs.

Factor Function	Factor Belief	Variable	Variable Belief
$f_{Y,r}\left(z_{r}\right)$	$b_{Y,r}\left(z_{r}\right)$	$z_{r}$	$q_{Z,r}\left(z_{r}\right)$
$f_{Z,r}\left(z_{r},h\right)$	$b_{Z,r}\left(z_{r},h\right)$	$h_{j}$	$q_{H,j}\left(h_{j}\right)$
$f_{H,j}\left(h_{j},\alpha_{k},s_{j},g_{j}\right)$	$b_{H,j}\left(h_{j},\alpha_{k},s_{j},g_{j}\right)$	$h_{j}$	$q_{H,j}\left(h_{j}\right)$
$f_{A,k}\left(\alpha_{k}\right)$	$b_{A,k}\left(\alpha_{k}\right)$	$\alpha_{k}$	$q_{A,k}\left(\alpha_{k}\right)$
$f_{S,d,l,k}\left(s_{d,l,k},s_{d-1,l,k}\right)$	$b_{S,d,l,k}\left(s_{d,l,k},s_{d-1,l,k}\right)$	$s_{d,l,k}$	$q_{S,d,l,k}\left(s_{d,l,k}\right)$
$f_{G,j}\left(g_{j},\omega_{j}\right)$	$b_{G,j}\left(g_{j},\omega_{j}\right)$	$g_{j}$	$q_{G,j}\left(g_{j}\right)$
$f_{\Omega ,j}\left(\omega_{j}\right)$	$b_{\Omega ,j}\left(\omega_{j}\right)$	$\omega_{j}$	$q_{\Omega ,j}\left(\omega_{j}\right)$

**Table 3:** Simulation parameter settings.

Parameter	Value	Parameter	Value
Carrier Frequency	2.6 GHz	OFDM Symbols *G*	3
Subcarrier Spacing $\Delta_{f}$	15 kHz	CP Length $N_{\mathrm{cp}}$	36
Subcarriers $N_{c}$	512	Delay Grids *L*	36
Active Subcarriers *N*	300	Angle Grids *D*	2 ×M
Pilot Subcarriers *P*	50	Potential Users *K*	500

## Data Availability

The data collected in this research are available upon request.
